# Pre-harvest UV irradiation differentially modulates terpenoids and esters in tarragon (*Artemisia dracunculus* L.) for enhanced essential oil quality

**DOI:** 10.3389/fpls.2026.1883766

**Published:** 2026-08-06

**Authors:** Chunjing Hou, Haitao Meng, Jialu Yang, Yiming Sun

**Affiliations:** 1 Xinjiang Key Laboratory of New Energy and Energy Storage Technology, Xinjiang Institute of Technology, Aksu, China; 2 Xinjiang Institute of Technology, Aksu, China

**Keywords:** *Artemisia dracunculus L*., essential oil quality, esters, industrial crop, terpenoids, UV irradiation, volatile organic compounds

## Abstract

Tarragon (*Artemisia dracunculus* L.) is an aromatic herb of industrial significance, valued for its essential oil composition that determines its applications in the flavor, fragrance, and cosmetic industries. Although ultraviolet (UV) radiation is known to modulate plant secondary metabolism, its potential as a pre-harvest strategy to enhance the volatile profile of tarragon for industrial applications remains unexplored. The present study employed headspace gas chromatography-ion mobility spectrometry (HS-GC-IMS) and headspace solid-phase microextraction gas chromatography-mass spectrometry (HS-SPME-GC-MS) to characterize the treatment-specific effects of supplemental ultraviolet A (UV-A), ultraviolet B (UV-B), and their combination (UV-A+UV-B) on the volatile metabolome of tarragon. Through HS-GC-IMS and HS-SPME-GC-MS analyses, 57 and 169 volatile organic compounds (VOCs) were identified, respectively. Across all irradiation treatments, terpenoids constituted the predominant chemical class, accounting for 40–52% of the total volatile profile. Orthogonal partial least squares discriminant analysis (OPLS-DA) yielded excellent discrimination among the four experimental groups (*Q*² > 0.95). Based on variable importance in projection (VIP) scores exceeding 1.0, a total of 21 differential VOCs were selected from the HS-GC-IMS dataset and 101 from the HS-SPME-GC-MS dataset, which collectively enabled clear separation of the four UV exposure regimens. UV-B irradiation specifically enhanced key industrial-relevant terpenoids (*γ*-terpinene, *β*-ocimene) and ketones, while combined UV-A+UV-B treatment synergistically increased ester levels, enriching compounds associated with fruity and floral notes desirable in perfumery applications. The complementary analytical platforms provided near-complete coverage of the tarragon volatilome, overcoming technique-specific limitations. These findings demonstrate that targeted pre-harvest UV irradiation differentially regulates terpenoids and esters, offering a practical strategy to improve the industrial quality of tarragon essential oil and providing a scientific basis for optimizing cultivation practices for aromatic crops.

## 1 Introduction

Tarragon (*Artemisia dracunculus L*.) is a perennial aromatic herb of the Asteraceae family, native to Eurasia and widely cultivated as an industrial crop for its essential oil, which is extensively utilized in the flavor, fragrance, and cosmetic industries (Ekiert et al., 2021). Its distinctive aromatic profile—characterized by herbal, anise-like, and slightly sweet notes—is highly valued for the production of natural flavorings, perfumery compounds, and aromatherapy products (Obolskiy et al., 2011). The characteristic aroma of tarragon is determined by its complex profile of volatile organic compounds (VOCs), primarily consisting of terpenoids, phenylpropanoids, aldehydes, ketones, and esters (Vârban et al., 2023). Among these, terpenoids such as *β*-ocimene, *γ*-terpinene, and methyl chavicol (estragole) have been identified as key contributors to the herb’s unique sensory attributes and are of particular interest for industrial essential oil extraction (Sahakyan et al., 2021).

Ultraviolet (UV) radiation is an environmental factor known to profoundly influence plant secondary metabolism (Shahzaidi et al., 2025). Although UV constitutes only a small fraction of solar radiation, it acts as a powerful elicitor of metabolic reprogramming, triggering the biosynthesis of a wide range of secondary metabolites including phenolics, flavonoids, and terpenoids (Crestani et al., 2024; Ghasemi et al., 2018; Khazaei et al., 2025; Kumar et al., 2025). UV-B (280–315 nm) and UV-A (315–400 nm) differentially regulate plant metabolic pathways due to their distinct penetration depths and perception modes (Verdaguer et al., 2017). UV-B is primarily perceived by the UVR8 photoreceptor and induces stress-related responses, often leading to the accumulation of protective compounds such as flavonoids and Terpenoids (J. Chen et al., 2023; Shamala et al., 2020). UV-A, while less energetic, penetrates deeper into leaf tissues and can modulate photosynthesis and secondary metabolism through cryptochrome-mediated signaling (Badmus et al., 2022; Zhang et al., 2020). Combined UV-A and UV-B effects are complex and may involve synergistic or antagonistic interactions (Cunningham et al., 2024; H. Liu et al., 2025; Mariz-Ponte et al., 2019; Qian et al., 2023; Vanhaelewyn et al., 2020).

The potential of UV irradiation as a pre-harvest tool to improve the quality of aromatic and medicinal plants has gained increasing attention in recent years. Several studies have demonstrated that supplemental UV exposure can enhance the accumulation of volatile compounds responsible for the aromatic properties of herbs and spices (Campos-Arguedas et al., 2022; Cvetkova et al., 2025; Gu et al., 2024; Juan-Polo et al., 2022; León-Chan et al., 2017). UV-B irradiation has been shown to increase monoterpene content in sweet basil (*Ocimum basilicum*) and to modulate the volatile profile of lemon balm (Ali et al., 2025; Bertoli et al., 2013; Ghasemzadeh et al., 2016; Semenova et al., 2022). In grape berries, UV-B treatment enhanced the production of monoTerpenoids, contributing to improved wine aroma (Del-Castillo-Alonso et al., 2020). More recently, UV-A has been recognised for its ability to influence volatile biosynthesis, with studies reporting increased levels of aldehydes and terpenoids in UV-A-exposed plants (Mumivand et al., 2021). Despite these advances, the impact of UV radiation on the volatile metabolome of tarragon—particularly the differential effects of UV-A, UV-B, and combined UV-A+UV-B—remains entirely unexplored. Given the economic importance of this industrial crop and the growing demand for natural aromatic ingredients, understanding how different UV wavelengths modulate its industrially relevant compounds is both timely and relevant.

Given that tarragon is rich in terpenoids and low-molecular-weight esters—compound classes for which HS-GC-IMS exhibits superior sensitivity—this platform was selected to complement conventional GC-MS analysis and to provide a rapid fingerprinting tool for comparing UV treatment-induced volatile variations. Owing to its low limit of detection, minimal sample preparation requirements, and ability to produce interpretable visual fingerprints of volatile compositions, headspace gas chromatography–ion mobility spectrometry (HS-GC-IMS) constitutes a rapid and highly sensitive approach for the detection of VOCs and has gained widespread adoption in volatilomics research (Ge et al., 2020; Parastar and Weller, 2024). Nevertheless, the headspace mode is known to have inherent analytical limitations, particularly regarding data reproducibility and susceptibility to matrix effects, which require careful experimental standardisation. In addition, its compound identification capability is constrained by the limited size of commercial databases. In contrast, headspace solid-phase microextraction gas chromatography–mass spectrometry (HS-SPME-GC-MS) provides high-resolution separation and reliable identification through mass spectral library matching, albeit with longer analysis times and lower sensitivity for some low-molecular-weight or highly polar compounds (Xu et al., 2024). The complementary use of these two platforms has recently been recognised as an effective strategy to achieve near-complete coverage of the plant volatilome, combining the sensitivity of IMS with the identification power of MS (Heting et al., 2020; Yao et al., 2024).

In the present study, a complementary analytical approach integrating HS-GC-IMS and HS-SPME-GC-MS was employed to comprehensively investigate the dynamic changes in the volatile metabolome of tarragon in response to supplemental UV-A, UV-B, and combined UV-A+UV-B irradiation. To ensure reproducibility and minimise variability, all samples were processed under strictly controlled conditions with internal standards and replicated measurements, as detailed in the Methods section. Although the chemical composition of tarragon has been investigated in several studies, comprehensive profiling of its volatile organic compounds (VOCs) remains limited. Existing research on tarragon has primarily focused on postharvest storage, essential oil nanoencapsulation, morphological diversity of wild populations and phenotypic relationships, polyphenol content, and the effects of light quality on bioactive compounds (Al-Zobaidi et al., 2026; Mehrabi et al., 2026; Qi et al., 2025; Sharif Abadi et al., 2026). By contrast, the volatile metabolome of tarragon—particularly its dynamic responses to environmental stress factors—has received considerably less attention.

Ultraviolet (UV) radiation is known to exert profound regulatory effects on plant secondary metabolism across diverse species. For example, UV-B irradiation has been shown to enhance the synthesis of volatile compounds in *Artemisia argyi* leaves (Gu et al., 2024). The combination of photosynthetically active radiation with UV-A and UV-B has been reported to modulate growth, antioxidant capacity, and essential oil composition in geranium (Jadidi et al., 2023). Despite these advances in other species, the relationship between UV radiation and volatile organic compounds has not been systematically investigated in tarragon. Furthermore, the combined use of complementary analytical platforms such as electronic nose, GC-MS, and GC-IMS for comprehensive VOC profiling of tarragon has rarely been reported. Multivariate statistical approaches, including OPLS-DA, have not been applied to identify differential volatile markers in tarragon under different UV treatments. These knowledge gaps warrant a systematic investigation into the UV-mediated regulation of the tarragon volatilome, which is the focus of the present study.

To the best of the authors’ knowledge, this study provides the first comprehensive volatilomic characterization of this industrially important aromatic crop under targeted UV irradiation. Specifically, the results reveal that UV wavelength differentially regulates terpenoid and ester biosynthesis, with combined UV-A+UV-B eliciting unique ester-enrichment responses not predictable from single-wavelength exposures. The identification of robust biomarkers further enables the establishment of a UV wavelength-specific metabolic fingerprint for practical quality monitoring applications.

The specific objectives of this study were: (I) to characterise the VOC profiles of tarragon under different UV treatments; (II) to identify differential volatile markers that discriminate the irradiation regimes using multivariate statistics (OPLS-DA); and (III) to elucidate the differential regulatory effects of UV wavelengths on key industrial-relevant compound classes, particularly terpenoids and esters. It was hypothesised that UV-B and UV-A+UV-B treatments would differentially enhance specific groups of VOCs, thereby modulating the overall aromatic profile of the tarragon essential oil. The findings of this study are expected to provide a scientific basis for the development of targeted pre-harvest UV irradiation strategies to improve the industrial quality of tarragon and other aromatic crops, and to offer a methodological framework for the volatilomic analysis of industrial crops.

## 2 Materials and methods

### 2.1 Plant materials and growth conditions

Tarragon (*Artemisia dracunculus* L.) plants were collected from Wensu County (41°N, 80°E), Aksu Prefecture, Xinjiang Uygur Autonomous Region, China. This region is characterised by an elevation gradient of 1,500–3,500 m and a mean annual temperature of 7.8 °C. The original wild soil collected from the same habitat was air-dried, homogenized, and passed through a 5-mm sieve to remove stones and plant debris to ensure uniformity. Each plastic pot (20 cm diameter × 18 cm height) was filled with 2.5 kg of the homogenized soil.

Uniformly growing plants of similar size and growth stage were selected from field conditions and transplanted into individual plastic pots containing the homogenized wild soil. A total of twelve potted plants were randomly assigned to four experimental groups (DZ, UV-A, UV-B, and UV-A+UV-B), with three biological replicates per treatment (each replicate consisting of one potted plant). Plants were irrigated regularly to maintain soil moisture at approximately 70% of field water-holding capacity throughout the experiment. After transplantation, plants were allowed to acclimate to greenhouse conditions for 7 days before UV treatment commenced. When new shoots reached approximately 5 cm in length, plants were subjected to UV treatments as described below. Supplemental UV irradiation was applied daily within the natural photoperiod (08:30 to 20:30), providing a daily exposure duration of 12 h.

Following UV exposure, above-ground parts (stems and leaves) were harvested simultaneously from all treatment groups between 09:00 and 10:00 to minimise diurnal variation. The harvested plant material was rinsed with distilled water to remove surface dust, then air-dried to constant weight at ambient temperature (25 ± 2 °C) in a well-ventilated room. The drying process was monitored by daily weighing until no further weight change was observed. The dried samples were ground to a homogeneous powder using a laboratory mill (particle size< 0.5 mm) and stored in airtight containers at 4 °C until further analysis.

### 2.2 UV irradiation treatments

To evaluate the effects of UV radiation on tarragon volatile profiles, four different UV treatment regimes were established: (1) Ambient UV (DZ): plants received only natural sunlight without any supplemental UV irradiation; (2) Supplemental UV-A (UV-A): plants were exposed to supplemental UV-A radiation using UV-A-340 lamps (40 W, 120 cm, Q-Lab Corporation, USA) with an emission peak at 340 nm; (3) Supplemental UV-B (UV-B): plants were exposed to supplemental UV-B radiation using UV-B-313 lamps (40 W, 120 cm, Q-Lab Corporation, USA) with an emission peak at 313 nm; (4) Combined UV-A+UV-B (UV-A+B): plants were exposed simultaneously to both supplemental UV-A and UV-B radiation sources, with intensities maintained at the same levels as in the individual treatments. The schematic diagram of the experimental apparatus and the four UV treatment regimens (DZ, UV-A, UV-B, and UV-A+UV-B) is presented in **Supplementary Figure 1**.

All supplemental UV treatments were applied daily within the natural photoperiod (08:30 to 20:30), providing a daily exposure duration of 12 h. Treatments commenced when plants reached approximately 5 cm in height and were continued for 15 consecutive days until plants reached the flowering stage. Control plants (DZ) were placed under the same experimental conditions but shielded from supplemental UV radiation using UV-blocking filters.

### 2.3 HS-GC-IMS analysis of VOCs

VOCs in tarragon samples were quantified via HS-GC-IMS. The analytical procedure was adapted from a previously reported method with minor adjustments (Fu et al., 2022). A FlavourSpec^®^ GC-IMS system (Gesellschaft für Analytische Sensorsysteme mbH, Dortmund, Germany) interfaced with an automatic headspace sampler (CTC Analytics AG, Zwingen, Switzerland) served as the analytical platform. Dried tarragon material (1.0 g) was precisely weighed, transferred into a 20 mL headspace vial, and crimp-sealed. All sample preparations and measurements were conducted in triplicate.

The headspace sampling parameters were adapted from the method of (Ratseewo et al., 2025) with minor modifications based on preliminary tests performed on tarragon samples to ensure optimal extraction efficiency for this specific plant matrix. The established parameters included an incubation temperature of 60 °C for 20 min under agitation at 500 rpm, a splitless injection volume of 500 μL, and an injector needle temperature of 85 °C. Separation was accomplished on an MXT-5 capillary column (15 m × 0.53 mm i.d., 1 μm film thickness) held at 60 °C, with ultra-high purity nitrogen (≥99.999%) as the mobile phase. The carrier gas flow rate was programmed as follows: 2.0 mL·min^-^¹ for the first 2 min, then linearly increased to 10.0 mL min^-^¹ over 8 min, further ramped to 100.0 mL·min^-^¹ over 10 min, and finally to 150.0 mL·min^-^¹ over 10 min, resulting in a total run time of 30 min. The GC inlet was maintained at 80 °C.

Ionization was performed using a ³H tritium source. The IMS conditions comprised a drift tube length of 53 mm, an electric field strength of 500 V·cm^-^¹, a drift tube temperature of 45 °C, and a drift gas (high-purity nitrogen, ≥99.999%) flow rate of 75 mL·min^-^¹, with positive-ion mode detection throughout. A six-ketone standard mixture was analyzed to establish retention time and retention index (RI) calibration curves; RI values for detected compounds were subsequently derived from their retention times. Compound identification was achieved by matching the experimental RI and ion drift time against the NIST 2020 GC-RI database and the IMS drift time library integrated into the Vocal software. Data acquisition and processing were performed using the Vocal platform, and the built-in plugins (Reporter and Gallery Plot) were employed to generate three-dimensional and two-dimensional spectra, difference spectra, and volatile fingerprints, enabling cross-sample comparative profiling of VOCs.

### 2.4 HS-SPME-GC-MS analysis of VOCs

A previously published methodology, subjected to minor modifications, was adopted for the complementary determination of VOCs via headspace solid-phase microextraction coupled with gas chromatography–mass spectrometry (HS-SPME-GC-MS) (Geng et al., 2024; Guan et al., 2026; H. Huang et al., 2024; Yiwen Zhang et al., 2022; Zhong et al., 2024; F. Zhou et al., 2025; L. Zhou et al., 2025). Dried tarragon samples (1.0 g) were accurately weighed into 15 mL sealed extraction vials and immediately sealed with PTFE-silicone septa. The extraction of VOCs utilized a 2-cm SPME fiber bearing a 50/30 μm layer of divinylbenzene/carboxen/polydimethylsiloxane (DVB/CAR/PDMS, Supelco, Bellefonte, PA, USA). Prior to analysis, the fiber was thermally conditioned by placing it in the GC-MS injector at 250 °C for a duration of 30 min, consistent with the supplier’s specifications.

Extraction procedure: Each sample vial was placed on a combined heating and stirring module and equilibrated at 70 °C under stirring at 1000 rpm for 15 min. Following equilibration, the SPME fiber was introduced into the vial headspace, positioned approximately 1.0 cm above the sample surface, and maintained there for 40 min to facilitate VOC adsorption. After extraction, the fiber was promptly retracted, withdrawn from the vial, and then inserted into the GC-MS injection port, where thermal desorption was performed at 250 °C for 3 min in splitless mode.

GC-MS analysis: Separation and identification of VOCs were conducted using an Agilent 7890A gas chromatograph coupled with an Agilent 5975C mass spectrometer (Agilent Technologies, Santa Clara, CA, USA). Chromatographic separation was performed on an HP-INNOWAX capillary column (60.0 m × 250 μm × 0.25 μm film thickness, Agilent Technologies). The column oven temperature program was: initial temperature 40 °C held for 2 min, then raised to 120 °C at a rate of 5 °C·min^-^¹, subsequently increased to 230 °C at 10 °C·min^-^¹, and finally held for 10 min. The injector and transfer line temperatures were set to 250 °C and 240 °C, respectively. Helium (purity ≥ 99.999%) was used as the carrier gas at a constant flow rate of 1.0 mL·min^-^¹.

Mass spectrometry conditions: The mass spectrometer was operated in electron ionization (EI) mode at 70 eV. The ion source temperature was maintained at 230 °C, and the quadrupole temperature at 150 °C. Mass spectra were recorded over a scan range of m/z 20–500 at a scan rate of 2.7 scans·s^-^¹. Compound identification was achieved by matching the acquired mass spectra against the NIST23 library and by comparing retention indices with those of authentic standards or reported values. Peak integration was performed using Agilent MSD ChemStation software (version E.02.02). Relative quantification was carried out using the area normalization method, and the results are reported as relative peak area percentages (i.e., individual peak area relative to total peak area).

Compound identification was performed by matching mass spectra against the NIST23 library and cross-referencing the calculated retention indices (RI) with those reported in the NIST database and literature, as recommended for complex volatile mixtures (de Oliveira et al., 2024; Kakanopas et al., 2022; von Mühlen and Marriott, 2011; Williams and Buica, 2020).

The relative contents of volatile compounds were calculated using the area normalization method (peak area of individual compound/total peak area of all identified volatiles). While we acknowledge that authentic standards were not available for all compounds, this relative quantification approach is widely employed in comparative volatilomics studies to evaluate treatment-induced changes, as the primary objective of this study was to compare the relative abundance of volatile compounds among UV treatment groups rather than to determine absolute concentrations. The consistency between HS-GC-IMS and HS-SPME-GC-MS results further supports the reliability of the observed trends.

### 2.5 Data processing and statistical analysis

For the visualization and comparative evaluation of volatile profiles obtained by HS-GC-IMS, two dedicated plugins integrated into the Vocal software—namely Reporter and Gallery Plot—were employed. These tools enabled the generation of three-dimensional spectra, two-dimensional topographic maps, difference spectra, and fingerprint plots, facilitating cross-sample comparisons of volatile organic compound profiles.

Relative quantification of compounds detected by HS-SPME-GC-MS was carried out using the area normalization method. Under this approach, the relative content of an individual compound (denoted as C_i_) was derived from the ratio of its peak area (A_i_) to the total integrated peak area of all identified volatile components, expressed as a percentage according to the following equation:


$${\text{C}}_{\text{i}}=\frac{{\text{A}}_{\text{i}}}{{\text{A}}_{1}+{\text{A}}_{2}+\cdots +{\text{A}}_{\text{i}}+\cdots +{\text{A}}_{\text{n}}}\times 100\%$$

Where n represents the total number of identified compounds.

Each experiment was carried out in triplicate using biologically independent samples. All data are presented as mean values with their corresponding standard deviations (SD). To determine statistically significant differences among the treatment groups, a one-way analysis of variance (ANOVA) was conducted, followed by Duncan’s multiple range post-hoc test. These statistical analyses were performed using IBM SPSS Statistics (version 25.0; IBM Corp., Armonk, NY, USA). A probability value of *p* < 0.05 was considered to indicate statistical significance.

For multivariate data analysis, orthogonal partial least squares discriminant analysis (OPLS-DA) was carried out using SIMCA software (version 14.0, Umetrics, Umeå, Sweden). The independent variable matrix *X* was arranged with dimensions n × p, where n represents the number of samples (biological replicates) and p represents the number of volatile compound features (variables). The dependent matrix *Y* was organized as *n* × *k*, where *k* denotes the number of class categories (*k* = 4 for the four UV treatment groups in this study: DZ, UV-A, UV-B, and UV-A+UV-B). All samples included in the modeling had complete data with no missing values.

Prior to OPLS-DA modeling, the data matrix was mean-centered and subjected to unit variance scaling (UV-scaling) to eliminate dimensional differences among compound features. The OPLS-DA algorithm decomposes the variation in the *X* matrix into one predictive component (*t*_p_) correlated with *Y* and one or more orthogonal components (*t*_o_) uncorrelated with *Y*. The optimal number of orthogonal components was determined by 7-fold cross-validation to prevent overfitting. Model validity was evaluated by *R*²X (cumulative modeled variation in *X*), *R*²Y (cumulative modeled variation in *Y*), and *Q*² (cumulative predicted variation) values. Variable importance in projection (VIP) values were calculated to identify the most discriminative compounds, with VIP > 1.0 considered as significant contributors to group separation. To further confirm that the model was not obtained by random overfitting, 200 permutation tests were performed by randomly shuffling the class labels and comparing the *R*²*Y* and *Q*² values of the permuted models with those of the original model; *Q*² intercept values below zero were taken as evidence of model validity.

Hierarchical clustering analysis (HCA) was performed using TBtools software (version 1.098) to generate heatmaps visualising the relative abundance patterns of key discriminatory VOCs across different treatment groups.

Hierarchical clustering analysis (HCA) is a widely employed unsupervised clustering algorithm in multivariate statistical analysis that effectively reduces the dimensionality of raw monitoring data and significantly simplifies the complex structure of high-dimensional datasets. In this study, HCA was performed to enable cluster-based visualization of the volatile profiles across samples. The resulting heatmaps present the peak volume differences of each volatile compound through color gradients, with red indicating higher relative abundance and blue indicating lower relative abundance. Samples grouped within the same cluster are considered to share a high degree of similarity and association. Specifically, Euclidean distance was used to quantify the dissimilarity among samples, with smaller Euclidean distance values indicating greater similarity in volatile composition between the corresponding samples. This clustering approach provides an intuitive and statistically robust means of identifying treatment-specific metabolic signatures and complementing the supervised OPLS-DA classification.

## 3 Results

### 3.1 Comprehensive volatile profiling reveals treatment-specific responses to UV irradiation

**Figure 1:**
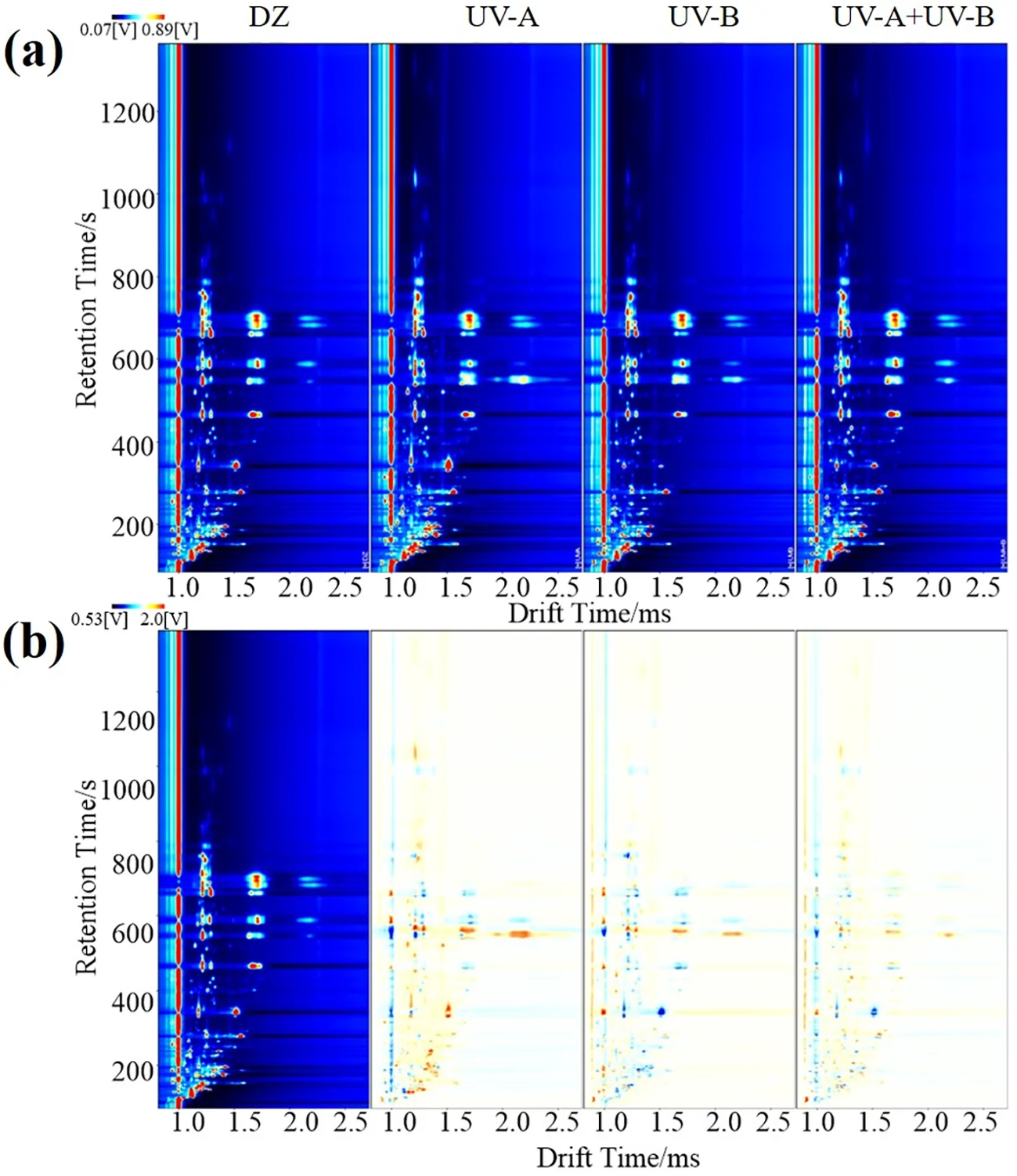
HS-GC-IMS spectra of tarragon under different ultraviolet irradiation. **(a)** 2D-topographic spectra, **(b)** differential spectra with DZ as the reference for comparison results. DZ: ambient UV control; UV-A: supplemental UV-A treatment; UV-B: supplemental UV-B treatment; UV-A+UV-B: combined UV-A and UV-B treatment.

The volatile organic compound profiles of tarragon under distinct UV irradiation regimens were preliminarily characterized using HS-GC-IMS. To enable direct visual comparison among groups, the acquired three-dimensional spectral data were transformed into two-dimensional topographic plots (presented in **Figure 1a**). In these plots, the normalized ion drift time is displayed on the x-axis, while the gas chromatographic retention time is shown on the y-axis; each resolvable spot corresponds to an individual volatile compound. The abundance of each compound is encoded by color, with red indicating higher signal intensity and blue denoting lower intensity. The majority of detected volatiles fell within retention time windows of 50–800 s and drift time ranges of 1.0–2.0 ms.

To highlight treatment-induced differences, the spectrum of the ambient UV control (DZ) was used as a reference for background subtraction, generating differential spectra for the UV-A, UV-B, and UV-A+UV-B treatment groups (**Figure 1b**). A color-based scheme was adopted for the differential plots: white denotes no concentration difference compared with the control; red indicates a statistically significant increase in the treatment group; and blue indicates a statistically significant decrease. This visualisation strategy effectively revealed the differential accumulation patterns of volatile compounds induced by each UV regime, providing an intuitive overview of treatment-specific metabolic responses.

**Figure 2:**
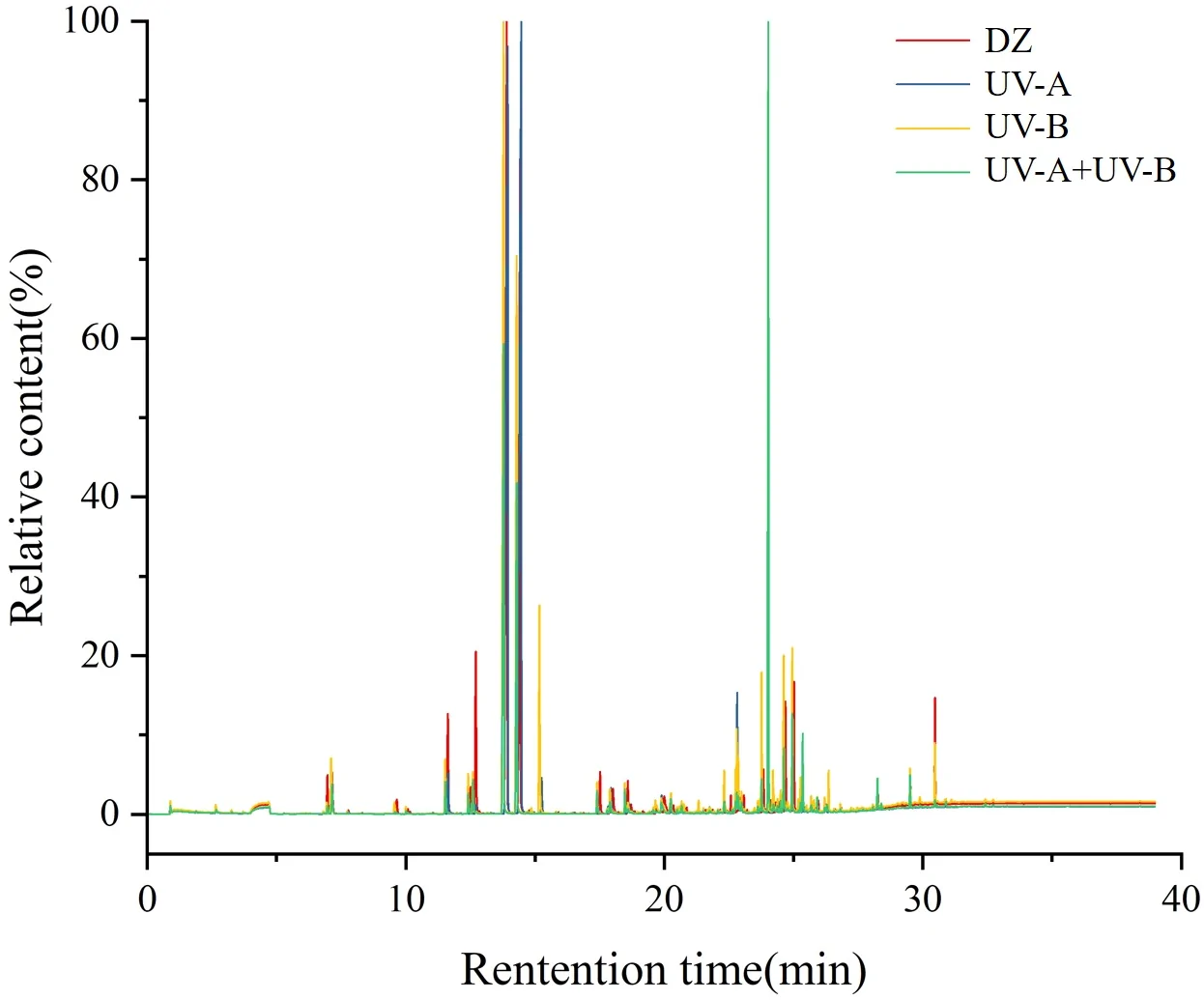
Total ion chromatograms of VOCs from tarragon under different ultraviolet irradiation obtained by HS-SPME-GC-MS. DZ: ambient UV control; UV-A: supplemental UV-A treatment; UV-B: supplemental UV-B treatment; UV-A+UV-B: combined UV-A and UV-B treatment.

To obtain complementary and more comprehensive volatile profiles, all samples were analysed by HS-SPME-GC-MS. The total ion chromatograms (**Figure 2**) revealed substantial differences in both the number and intensity of volatile peaks among treatment groups, consistent with the HS-GC-IMS findings. However, the specific compounds showing maximal responses differed between the two analytical platforms. For example, while HS-GC-IMS indicated highest (*E*)-2-hexenal abundance under UV-A irradiation, the TIC profiles showed that estragole (retention time 24–25 min) was most abundant under combined UV-A+UV-B treatment, whereas *trans*-*β*-ocimene (12.5 min) was lowest under UV-B and highest under UV-A. These discrepancies highlight the complementary nature of the two techniques, as they operate on different detection principles and exhibit varying sensitivities toward different compound classes (Heting. Qi et al., 2020; Yao et al., 2024).

### 3.2 Complementary identification expands the known volatilome of tarragon

**Figure 3:**
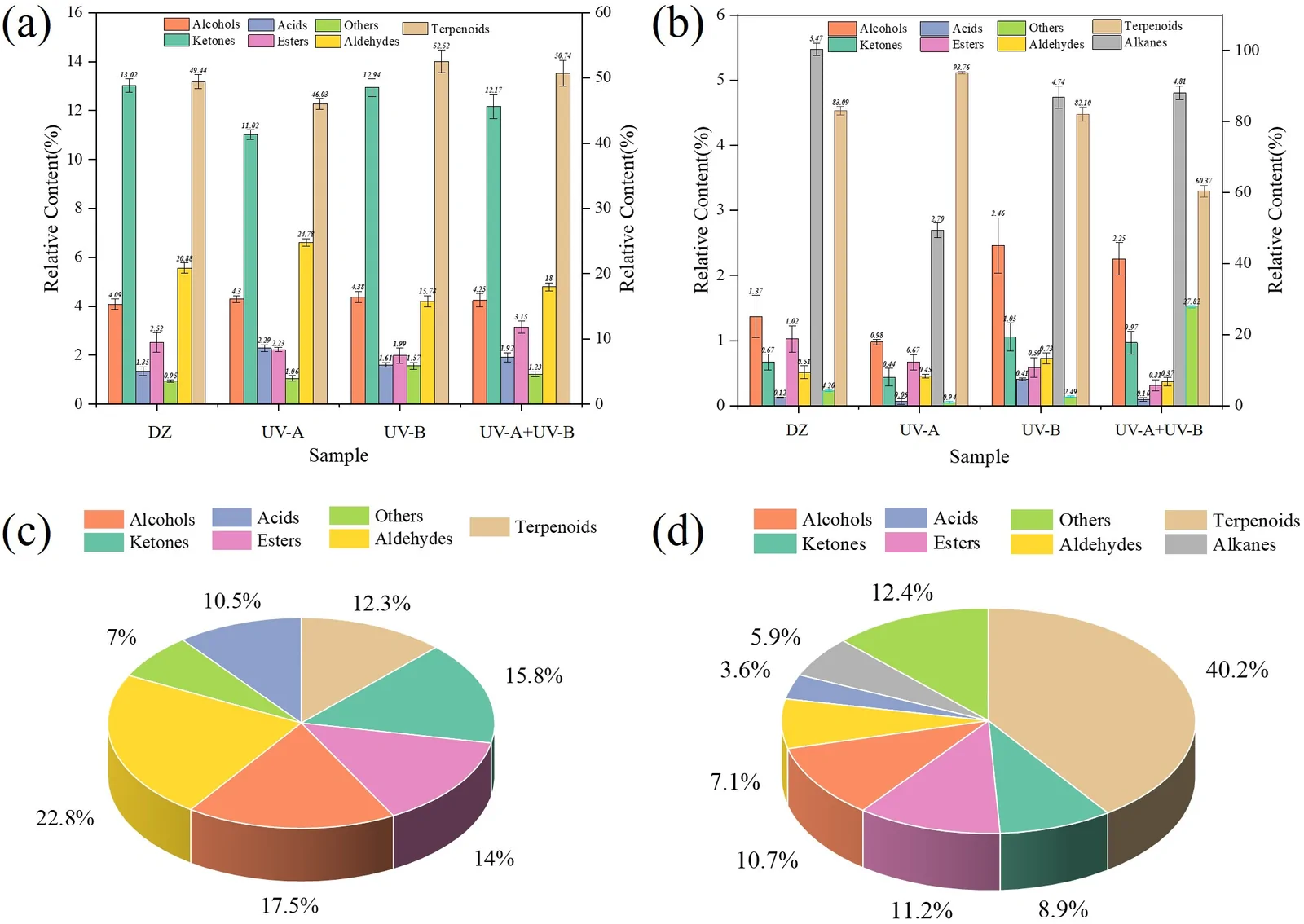
Relative content and compositional ratio of volatile organic compound classes in tarragon under different ultraviolet treatments. **(a)** Relative content from HS-GC-IMS dataset, **(b)** Relative content from HS-SPME-GC-MS dataset, **(c)** Compositional ratio from HS-GC-IMS dataset, **(d)** Compositional ratio from HS-SPME-GC-MS dataset. DZ: ambient UV control; UV-A: supplemental UV-A treatment; UV-B: supplemental UV-B treatment; UV-A+UV-B: combined UV-A and UV-B treatment.

A total of 117 volatile peaks were detected across all samples by HS-GC-IMS, of which 57 compounds were unambiguously identified based on retention indices and drift time matching with the GC-IMS library (**Supplementary Table 1**). The identified compounds included 7 Terpenoids, 10 alcohols, 13 aldehydes, 9 ketones, 8 esters, 4 acids, and other 6 substances, which accounted for 12.3%, 17.5%, 22.8%, 15.8%, 14%, 7%, and 10.5% of the total compounds, respectively (**Figure 3c**). The remaining 21 peaks could not be identified due to limitations of the current database, highlighting one of the inherent constraints of HS-GC-IMS technology (Yao et al., 2024). It is noteworthy that in ion mobility spectrometry, monomer ions can form complexes with neutral molecules during drift, resulting in multiple signals (monomers, dimers, and polymers) for a single compound (Chen et al., 2020). This phenomenon is particularly pronounced for Terpenoids at high concentrations, which may appear as three or even four distinct peaks. While HS-GC-IMS is primarily considered a qualitative technique (Xiao et al., 2022), peak volume conversion to relative content enables reliable comparative analysis of volatile differences among samples.

HS-SPME-GC-MS analysis identified a total of 169 volatile compounds across the four treatment groups (**Supplementary Table 2**), comprising 68 Terpenoids, 15 ketones, 19 esters, 18 alcohols, 12 aldehydes, 6 acids, 10 alkanes, and 21 other compounds. Terpenoids were the dominant class (40.2% of total volatiles), followed by esters (11.2%), alcohols (10.7%), and ketones (8.9%), collectively accounting for over 70% of all VOCs (**Figure 3d**). These compound classes are well-established contributors to the woody, floral, and fruity aroma notes characteristic of aromatic plants used in the fragrance industry (Wang et al., 2025). In terms of compound numbers, 115 compounds were detected in UV-A and UV-B groups, 98 in the combined UV-A+UV-B group, and 102 in the DZ control, suggesting that combined UV treatment may promote greater volatile diversity.

Qualitative analysis based on peak volumes revealed that terpenoids were the predominant chemical class across all treatment groups, accounting for 46.03–52.52% of total volatiles (**Figure 3a**). Aldehydes constituted the second most abundant class (15.78–24.78%), followed by ketones (11.02–13.02%), alcohols (4.09–4.38%), esters (1.99–3.15%), acids (1.35–2.29%), and others (0.95–1.57%). HS-SPME-GC-MS qualitative comparison (**Figure 3b**) confirmed terpenoids as the dominant class (60.37–93.76%), followed by others (0.94–27.82%), alkanes (2.70–5.47%), alcohols (0.98–2.46%), ketones (0.44–1.05%), esters (0.31–1.02%), aldehydes (0.37–0.73%) and Acids (0.06–0.41%). Treatment-specific responses were evident: UV-B stress increased the relative content of terpenoids and alcohols; UV-A stress enhanced aldehydes and acids; ketones showed variable responses to UV-A; and esters were elevated under combined UV-A+UV-B treatment. These differential responses suggest that distinct UV wavelengths activate specific branches of volatile biosynthetic pathways, consistent with previous reports on wavelength-dependent metabolic regulation in plants (Verdaguer et al., 2017).

**Figure 4:**
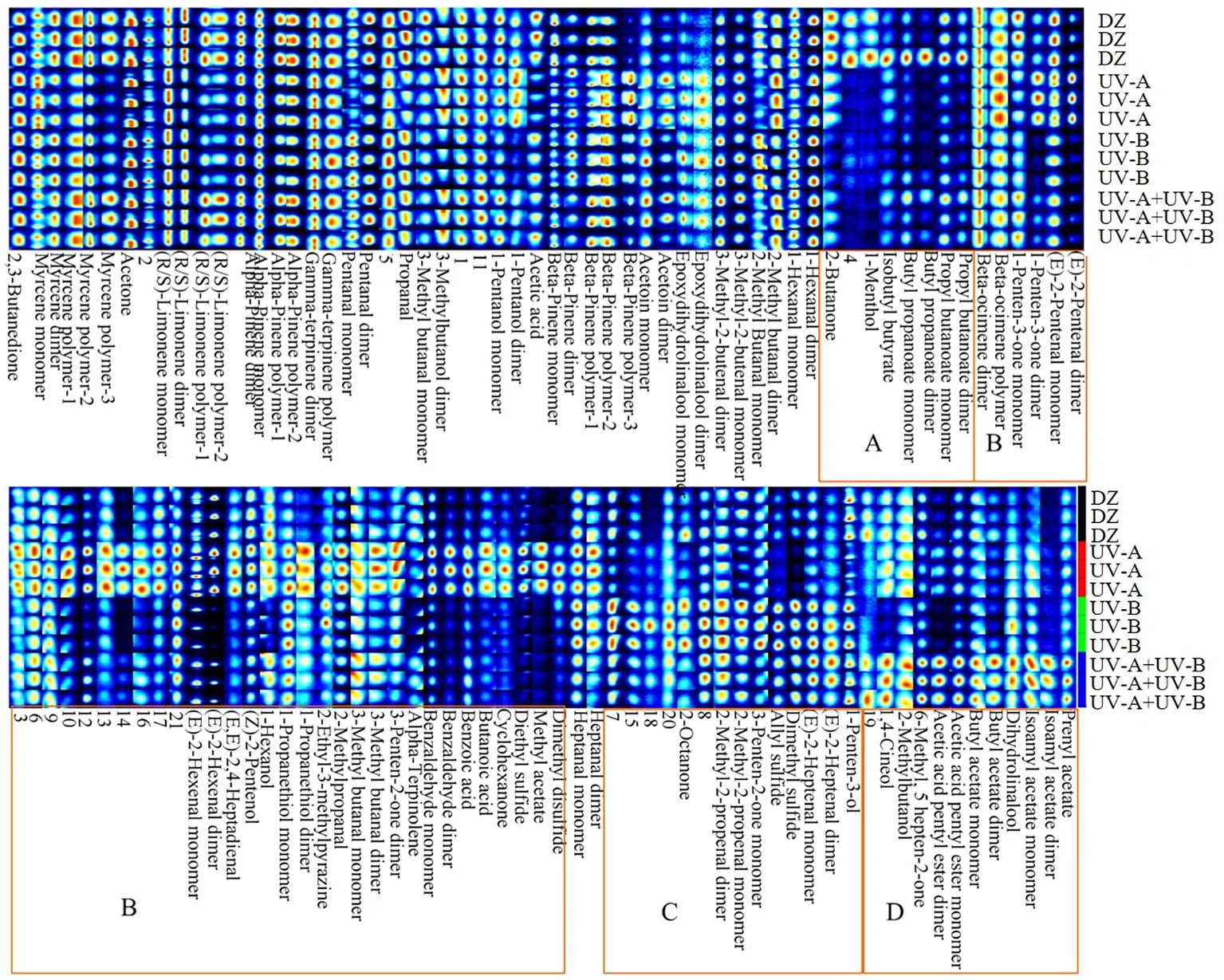
HS-GC-IMS fingerprint plot based on signal intensity of tarragon with different ultraviolet treatments. Each row represents a volatile compound, and each column represents a treatment group. Brighter colors indicate higher abundance. Regions **(A–D)** indicate compound clusters with distinct accumulation patterns as described in the text. DZ: ambient UV control; UV-A: supplemental UV-A treatment; UV-B: supplemental UV-B treatment; UV-A+UV-B: combined UV-A and UV-B treatment.

To comprehensively visualise treatment-induced changes, fingerprint analysis was performed on all HS-GC-IMS detected volatile compounds (**Figure 4**). This approach enables direct comparison of signal intensities across samples, with brighter colours indicating higher abundance (Guo et al., 2018; Yao et al., 2022). Based on accumulation patterns, the identified compounds were categorised into four distinct clusters. Region A comprised compounds with highest abundance in DZ samples, including 1-menthol, isobutyl butyrate, butyl propanoate, and propionic acid. Region B contained compounds maximally induced by UV-A treatment, such as *β*-ocimene, 1-penten-3-one dimer, (*E*)-2-pentenal, and (*E*)-2-hexenal. Region C featured compounds most abundant under UV-B, including 2-octanone, 2-methyl-2-propanal, and 3-penten-2-one. Region D consisted of compounds showing highest levels under combined UV-A+UV-B treatment, including 1,4-cineol, 2-methylbutyl alcohol, and pentyl acetate. These distinct accumulation patterns demonstrate that each UV regime elicits a unique metabolic signature, providing a basis for subsequent marker selection.

Comparison of the qualitative results obtained by the two analytical platforms revealed both consistencies and notable differences (**Supplementary Table 3**). Ten compounds were commonly identified by both techniques, but their relative abundances varied considerably. For example, (*E*)-*β*-ocimene ranged from 4.29% to 38.76% by HS-SPME-GC-MS but only 1.45% to 4.44% by HS-GC-IMS. Conversely, *γ*-terpinene showed concentrations of 0.01–0.03% by HS-SPME-GC-MS compared to 1.72–6.61% by HS-GC-IMS. These discrepancies reflect the fundamentally different detection principles of ion mobility spectrometry and mass spectrometry (Sorribes-Soriano et al., 2018). HS-GC-IMS exhibits higher sensitivity for certain compound classes, particularly dimers, terpenoids, and heterocyclic compounds, but its identification capability is constrained by database limitations. In the present study, several unidentified compounds in the HS-GC-IMS fingerprint (**Figure 4**) showed high abundance and treatment-specific responses, suggesting they may contribute importantly to tarragon aroma. Conversely, HS-SPME-GC-MS detected 169 compounds versus 57 by HS-GC-IMS, and identified several compounds not detected by IMS, including 2,3-butanedione, benzoic acid, pentyl acetate, (*E*)-2-hexenal, (*Z*)-2-pentenol, and epoxydihydrolinalool. These complementary strengths demonstrate that comprehensive volatilomic characterisation requires the integration of both analytical platforms (Heting et al., 2020; Yao et al., 2024).

### 3.3 Multivariate analysis discriminates UV treatments and identifies differential volatile markers

**Figure 5:**
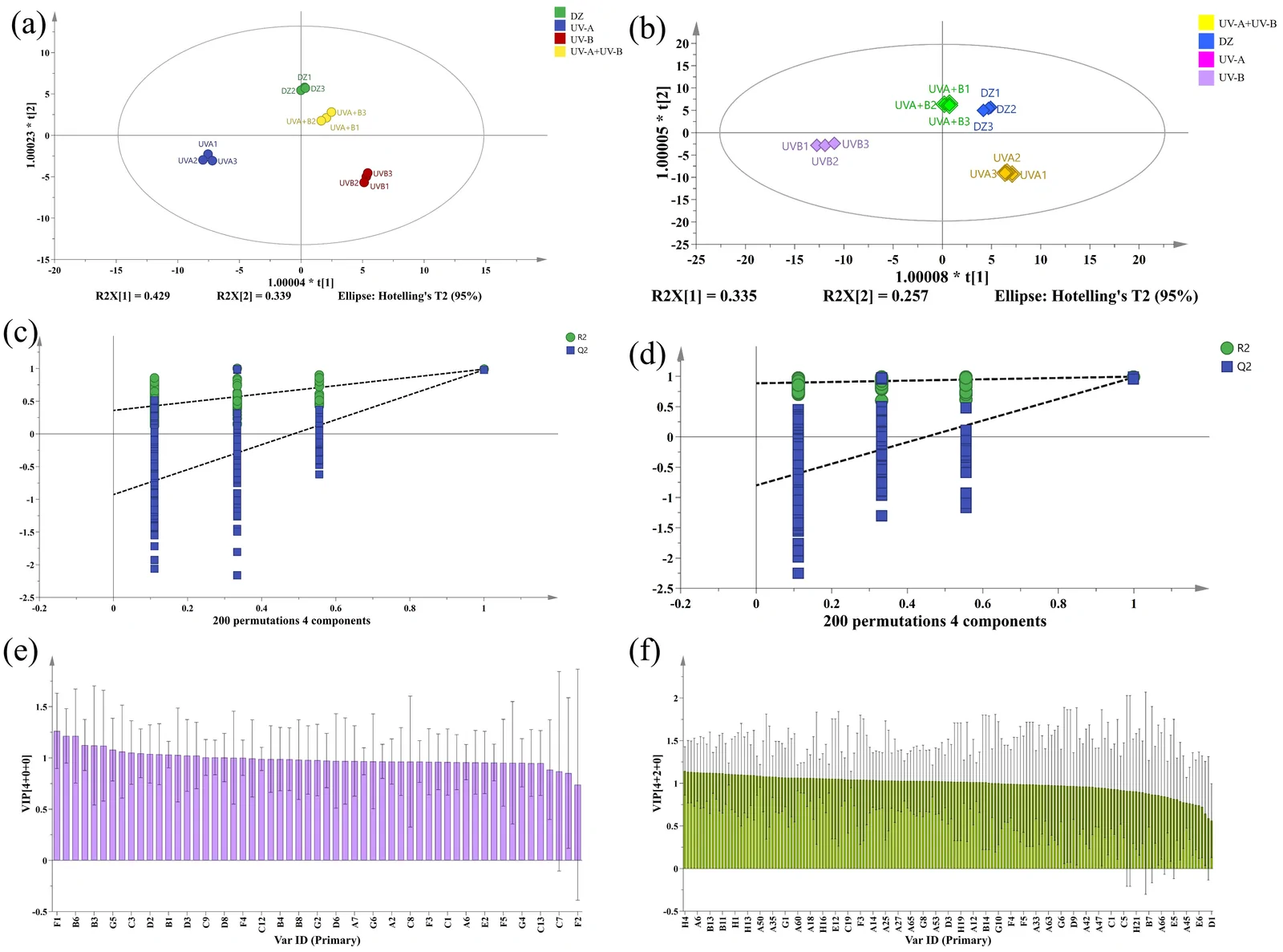
Orthogonal partial least squares-discriminant analysis (OPLS-DA) of tarragon volatile profiles under different ultraviolet treatments. **(a)** OPLS-DA score plot of HS-GC-IMS dataset, **(b)** OPLS-DA score plot of HS-SPME-GC-MS dataset, **(c)** cross-validation results of HS-GC-IMS model (200 permutation tests), **(d)** cross-validation results of HS-SPME-GC-MS model (200 permutation tests), **(e)** Variable importance in projection (VIP) scores of each variable from HS-GC-IMS dataset, **(f)** VIP scores of each variable from HS-SPME-GC-MS dataset. DZ: ambient UV control; UV-A: supplemental UV-A treatment; UV-B: supplemental UV-B treatment; UV-A+UV-B: combined UV-A and UV-B treatment.

To obtain a robust classification of samples across different UV exposure conditions, a supervised OPLS-DA model was constructed based on the two analytical datasets (HS-GC-IMS and HS-SPME-GC-MS). The choice of OPLS-DA is supported by its documented efficacy in metabolomics for sample discrimination and the construction of classification models (Y. Liu et al., 2023). The score plots derived from both datasets revealed clear separation among the four treatment groups (**Figures 5a, b**).

For the HS-GC-IMS dataset, the OPLS-DA model exhibited excellent goodness-of-fit and predictive capability, with *R*²*X* = 0.951, *R*²*Y* = 0.995, and *Q*² = 0.990 (**Figure 5a**). Volatile profiles of DZ and UV-A+UV-B samples clustered together in the first quadrant, indicating greater similarity between these two treatments, while UV-A samples concentrated in the third quadrant and UV-B samples in the fourth quadrant. Similarly, the model based on HS-SPME-GC-MS data showed strong performance (*R*²*X* = 0.907, *R*²*Y* = 0.996, *Q*² = 0.976), with DZ and UV-A+UV-B samples again clustering together in the first quadrant, UV-B in the third quadrant, and UV-A in the fourth quadrant (**Figure 5b**). Both *Q*² values substantially exceeded the acceptable threshold of 0.5 and approached 1.0, indicating high predictive accuracy and minimal overfitting risk. In OPLS-DA modelling, *R*²*X* and *R*²*Y* reflect model fit, while *Q*² indicates predictive capability (Bhumireddy et al., 2021; B.-M. Huang et al., 2018); values closer to 1.0 signify stronger model performance (Kandasamy et al., 2020; Ma et al., 2020).

To further validate model robustness and exclude overfitting, 200 permutation tests were conducted for both datasets (**Figures 5c, d**). The intercept values (HS-GC-IMS: *R*² = 0.335, *Q*² = -0.958; HS-SPME-GC-MS: *R*² = 0.885, *Q*² = -0.8) satisfied the criterion that *Q*² intercepts should be below zero, confirming the statistical validity of both models and the absence of overfitting (M. Liu et al., 2024). These results demonstrate that OPLS-DA based on volatile profiles can reliably discriminate tarragon samples subjected to different UV irradiation regimes.

To determine which volatile compounds were most influential in differentiating among treatments, variable importance in projection (VIP) values derived from OPLS-DA analyses were adopted as the selection criterion. VIP values quantify each variable’s contribution to the model, with variables having VIP > 1.0 generally considered to have above-average influence and to be statistically significant for group separation (How et al., 2023; Song et al., 2020; Tu et al., 2021). Applying this criterion, 21 compounds were selected from the HS-GC-IMS dataset and 101 compounds from the HS-SPME-GC-MS dataset as potential differential markers (**Supplementary Table 4**, **Figures 5e, f**). These markers, characterised by high discriminative power among treatment groups, can serve as candidate biomarkers for future targeted studies of UV effects on tarragon volatile metabolism.

**Figure 6:**
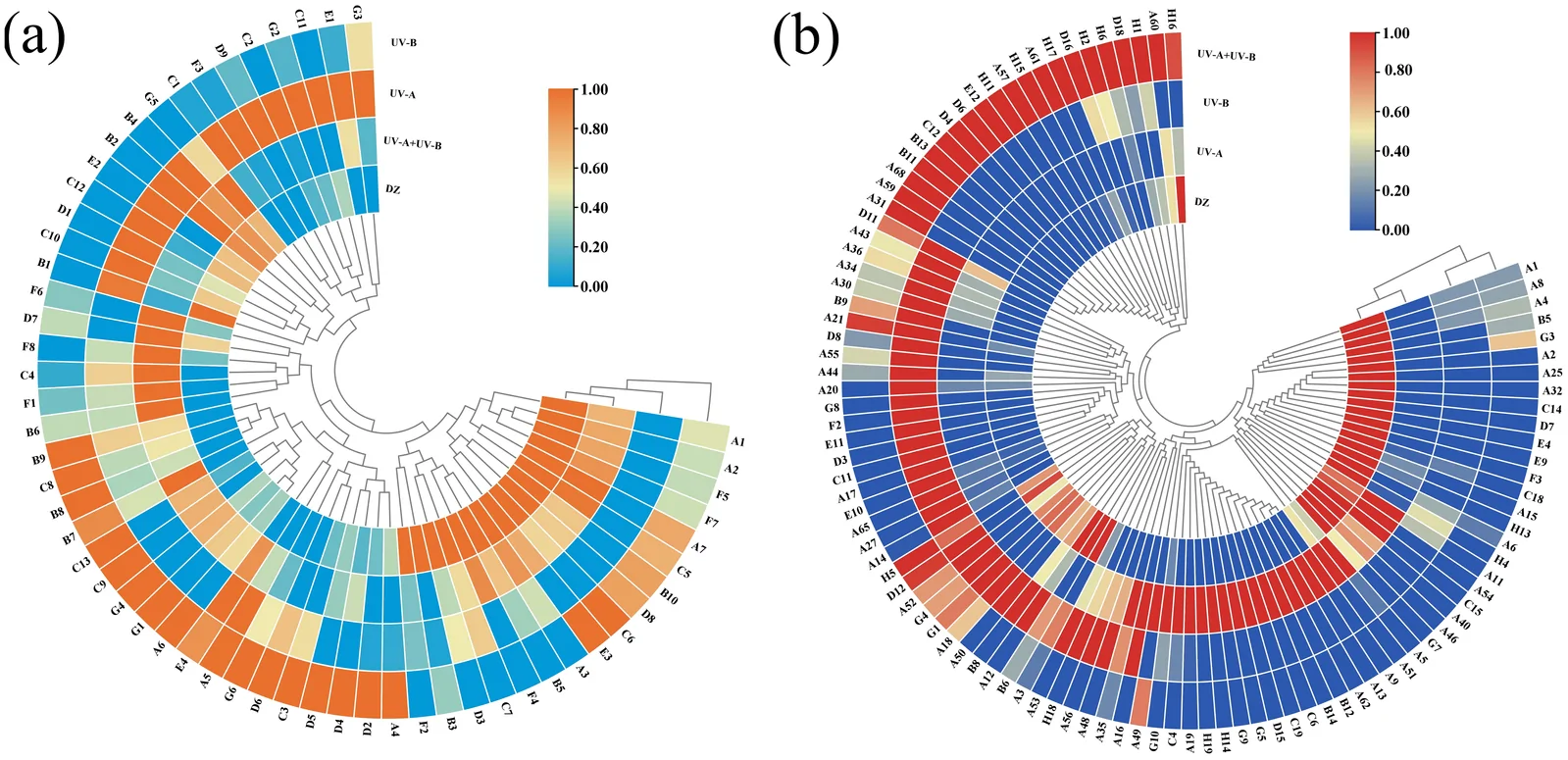
Hierarchical clustering analysis of tarragon samples based on relative abundance of key discriminatory VOCs. **(a)** HS-GC-IMS dataset, **(b)** HS-SPME-GC-MS dataset. Values represent normalized relative abundances (Z-score scaled) across samples. Color gradients represent relative abundance, with red indicating high abundance and blue indicating low abundance. DZ: ambient UV control; UV-A: supplemental UV-A treatment; UV-B: supplemental UV-B treatment; UV-A+UV-B: combined UV-A and UV-B treatment.

To further visualise the differential accumulation patterns of these markers, hierarchical clustering analysis was performed and the results were displayed as heatmaps (**Figures 6a, b**). Heatmaps use colour gradients to represent relative abundance, with red indicating high abundance and blue indicating low abundance (Wu et al., 2022). For the HS-GC-IMS dataset (**Figure 6a**), the four treatment groups clustered into three distinct categories: UV-A+UV-B and DZ formed one cluster, while UV-A and UV-B formed separate clusters. The close clustering of UV-A+UV-B and DZ suggests that combined UV treatment partially restores the volatile profile toward control conditions, possibly through interactive effects of the two wavelength bands. For the HS-SPME-GC-MS dataset (**Figure 6b**), clustering analysis grouped the four treatments differently: DZ and UV-A formed one cluster, while UV-A+UV-B and UV-B formed another. This discrepancy between the two analytical platforms likely reflects their differential sensitivities and selectivities, reinforcing the value of complementary approaches for comprehensive volatile profiling.

The HCA results further corroborated the treatment-specific metabolic signatures identified by OPLS-DA. As shown in **Figure 6a** (HS-GC-IMS dataset), UV-A+UV-B and DZ formed a single cluster, while UV-A and UV-B each formed distinct separate clusters. This clustering pattern indicates that the volatile profile under combined UV-A+UV-B treatment partially resembles that of the ambient control, suggesting that concurrent exposure to both UV wavelengths may elicit antagonistic effects that partially offset the individual metabolic perturbations induced by UV-A or UV-B alone. For the HS-SPME-GC-MS dataset (**Figure 6b**), DZ and UV-A clustered together, whereas UV-A+UV-B and UV-B formed another cluster. The discrepancy in clustering patterns between the two analytical platforms likely reflects their inherent differences in detection sensitivity and selectivity toward specific compound classes—HS-GC-IMS exhibits higher sensitivity for certain low-molecular-weight volatiles and their dimer/polymer forms, whereas HS-SPME-GC-MS provides broader coverage of the overall volatile metabolome. The Euclidean distance between clusters (as indicated by the dendrogram branch lengths) further supports these groupings: the relatively short branch lengths within each cluster confirm the high compositional similarity among biological replicates of the same treatment, while the longer branch lengths between clusters validate the treatment-specific divergence of volatile profiles. Importantly, the clustering patterns derived from the heatmaps are fully consistent with the OPLS-DA score plots (**Figures 5a, b**), demonstrating that both supervised and unsupervised multivariate approaches converge on the same conclusion—namely, and that UV wavelength is a critical determinant of volatile composition in tarragon.

### 3.4 UV wavelength differentially modulates key industrial-relevant chemical classes

Terpenoids showed generally high abundance across all samples, with specific compounds exhibiting treatment-specific responses. UV-B stress positively affected *γ*-terpinene and *β*-ocimene accumulation, consistent with the known role of UV-B in promoting terpene biosynthesis (Del-Castillo-Alonso et al., 2020). The HS-GC-IMS fingerprint (**Figure 4**, Region C) showed increased signals for several Terpenoids under UV-B, which was corroborated by HS-SPME-GC-MS qualitative data (**Figure 3b**). UV-A stress enhanced *β*-pinene, as observed in both platforms. Terpenoid content was highest in the UV-A group by HS-SPME-GC-MS, consistent with the known role of UV-A in activating terpene synthase genes and promoting terpenoid accumulation in medicinal and aromatic plants (Mumivand et al., 2021). The key terpenoids identified in this study, including (R/S)-limonene, isoamyl acetate, *γ*-terpinolene, *β*-ocimene, *β*-pinene, butyl acetate, and pentyl acetate, correspond well with the major volatile compounds previously reported for tarragon, such as terpinen-4-ol, *β*-ocimene, limonene, *trans*-anethole, *α*-phellandrene, and *β*-phellandrene (Majdan et al., 2020; Sahakyan et al., 2021).

Esters showed a unique response pattern. The HS-GC-IMS fingerprint (**Figure 4**, Region D) and the HS-SPME-GC-MS heatmap (**Figure 6b**) both indicated that compounds like isoamyl acetate, butyl acetate, and pentyl acetate were most abundant under the combined UV-A+UV-B treatment. The enhancement of esters under combined treatment suggests potential synergistic effects on ester biosynthesis pathways, which would enrich the fruity and floral notes of tarragon. Esters, particularly acetates and butyrates, are formed through the esterification of alcohols and acids and contribute significantly to fruity and floral sensory attributes. Ester content was highest in DZ controls and lowest under combined UV-A+UV-B treatment by HS-SPME-GC-MS qualitative analysis (**Figure 3b**), which appears contradictory to the fingerprint and heatmap findings. This discrepancy may reflect differences in detection sensitivity between the two platforms for specific ester compounds, or may indicate that while certain individual esters are enhanced by combined UV treatment, the total ester pool is reduced due to redirection of metabolic precursors toward other pathways under stress conditions.

Aldehydes including acetone, (*E*)-2-hexenal, 1-hexanal, and 2,3-butanedione were generally enhanced by UV treatments, consistent with UV-induced oxidative stress promoting lipid peroxidation and aldehyde formation. Both platforms consistently showed enhancement of aldehydes under UV treatments. For example, (*E*)-2-hexenal was highly induced by UV-A (HS-GC-IMS, **Figure 4**, Region B). Aldehydes, primarily generated through the oxidation of unsaturated fatty acids (Bi et al., 2023), were most abundant under UV-B treatment by HS-SPME-GC-MS qualitative analysis (**Figure 3b**). This observation aligns with the well-documented role of UV-B in inducing oxidative stress and lipid peroxidation, which can promote aldehyde formation (Gill and Tuteja, 2010).

Ketones also showed treatment-specific responses. Both platforms indicated enhancement of ketones under UV treatments, with HS-SPME-GC-MS showing highest ketone content under UV-B (**Figure 3b**). Ketones derive mainly from catabolic reactions of amino acids and from the thermal-oxidative decomposition of polyunsaturated fatty acids (Delcros et al., 2023), peaked under UV-B treatment. Among the ketones detected, *β*-ionone is particularly noteworthy for its contribution to the characteristic floral, violet-like aroma of *Artemisia* species. The HS-GC-IMS fingerprint (**Figure 4**, Region C) showed increased signals for ketones including 2-octanone, 2-methyl-2-propanal, and 3-penten-2-one under UV-B.

Alcohols were also modulated by UV treatments. HS-SPME-GC-MS qualitative analysis showed highest alcohol content under UV-B (**Figure 3b**). Alcohols—presumably formed via catabolic reactions of arachidonic and linoleic acids—were also maximally abundant under UV-B (He et al., 2020). Sensorially important examples included 1-octen-3-ol (mushroom and vanilla) and 2-ethyl-1-hexanol (rose-green, citrus, sweet) (Youjin Zhang et al., 2023).

In summary, the integrated analysis from both platforms reveals that UV radiation differentially regulates key aroma-contributing compound classes in tarragon. UV-B specifically enhances terpenoids (*γ*-terpinene, *β*-ocimene) and ketones, while combined UV-A+UV-B treatment promotes specific esters that enrich fruity and floral notes. Aldehydes are generally enhanced by UV treatments as markers of oxidative stress. The treatment-specific modulation of these key aroma contributors provides a scientific basis for developing targeted UV-based strategies to enhance tarragon flavour quality.

## 4 Discussion

### 4.1 UV wavelength-specific modulation of volatile profiles in an industrial aromatic crop

This study provides the first comprehensive volatilomic analysis of tarragon under targeted UV irradiation, revealing distinct metabolic signatures elicited by UV-A, UV-B, and combined UV-A+UV-B treatments. The clear separation observed in OPLS-DA models (*Q*² > 0.95) demonstrates that UV wavelength is a critical determinant of volatile composition in this industrial aromatic crop. These findings align with previous reports on wavelength-dependent metabolic regulation in plants (Lu et al., 2026; Verdaguer et al., 2017; Yoon et al., 2026) and extend them to an economically important species cultivated for essential oil production.

The predominance of terpenoids across all treatments (40–52% of total volatiles) underscores their central role in tarragon’s industrial value, as terpenoids constitute the primary aromatic components of essential oils used in perfumery, cosmetics, and natural flavorings (Sahakyan et al., 2021; Sharma et al., 2026; Yue et al., 2026). The differential accumulation patterns observed—UV-B enhancing *γ*-terpinene and *β*-ocimene, UV-A promoting *β*-pinene, and combined UV-A+UV-B stimulating esters—indicate that specific UV wavelengths activate distinct branches of terpenoid and ester biosynthesis pathways. This wavelength specificity offers a practical tool for tailoring essential oil composition to meet diverse industrial requirements.

### 4.2 UV-B treatment enhances industrially valuable terpenoids and ketones

The enhancement of γ-terpinene and β-ocimene under UV-B irradiation is consistent with the well-documented role of UV-B in promoting terpene biosynthesis through the activation of terpene synthase genes and the accumulation of protective secondary metabolites (Jin et al., 2025; Khazaei et al., 2025). γ-Terpinene is a key precursor of aromatic compounds such as thymol and carvacrol, which are highly valued in the fragrance and pharmaceutical industries (Mumivand et al., 2021). β-Ocimene, a major monoterpene in many essential oils, contributes to the fresh, floral notes desired in high-end perfumery (Campos-Arguedas et al., 2022; Gao et al., 2026). The increased abundance of these compounds under UV-B suggests that targeted UV-B exposure could be employed to produce tarragon essential oil with enhanced industrial value.

Similarly, the elevation of ketones under UV-B, including β-ionone, is noteworthy. β-Ionone is a norisoprenoid derived from carotenoid degradation and is prized for its violet-like aroma, widely used in fragrance formulations (Delcros et al., 2023; H. Fu et al., 2026). The UV-B-induced accumulation of such ketones may reflect increased oxidative stress and carotenoid cleavage, as UV-B is known to generate reactive oxygen species that promote apocarotenoid formation (Gill and Tuteja, 2010; R. Liu et al., 2026). These changes could be exploited to enrich tarragon essential oil with rare and expensive aroma compounds.

The HCA clustering patterns merit further biological interpretation. The close clustering of UV-A+UV-B and DZ in the HS-GC-IMS-based HCA (**Figure 6a**) suggests that simultaneous UV-A and UV-B exposure does not produce additive or synergistic effects on the volatile profile; instead, antagonistic interactions between the two wavelength bands may partially counteract the metabolic changes induced by each individual UV treatment. This observation aligns with previous reports that UV-A and UV-B can elicit opposing or competing regulatory effects on plant secondary metabolism through distinct photoreceptor-mediated signaling pathways—UV-A primarily acts through cryptochrome-mediated pathways, while UV-B signals via the UVR8 photoreceptor. The differential clustering patterns observed between the two analytical platforms further highlight the importance of integrating multiple detection techniques, as each platform captures a distinct subset of the volatile metabolome, and their combined use provides a more comprehensive view of treatment-induced metabolic reprogramming. The consistency between HCA and OPLS-DA results across both platforms reinforces the robustness of our classification and supports the conclusion that the observed treatment-specific volatile signatures are genuine metabolic responses rather than analytical artifacts.

### 4.3 Synergistic effects of combined UV-A+UV-B on ester biosynthesis

The combined UV-A+UV-B treatment uniquely enhanced specific esters, including isoamyl acetate, butyl acetate, and pentyl acetate, as evidenced by both HS-GC-IMS fingerprints and HS-SPME-GC-MS heatmaps. Esters are key contributors to fruity and floral notes in essential oils and are highly sought after in the fragrance industry for their pleasant and volatile nature (Bovio-Zenteno et al., 2026; Fang et al., 2026; Sun et al., 2026). The synergistic effect observed may result from the integration of UV-A-driven photosynthetic modulation and UV-B-induced stress responses, leading to increased availability of alcohol and acid precursors for esterification. This finding suggests that combined UV treatment could be used to produce tarragon essential oil with a more complex and desirable ester profile, catering to niche markets in perfumery.

However, it is noteworthy that total ester content measured by HS-SPME-GC-MS was highest in control samples, while specific esters were enhanced under combined treatment. This apparent discrepancy may reflect the differential sensitivity of the two analytical platforms or indicate metabolic reallocation under stress, where precursors are channeled toward a subset of esters rather than the total ester pool. Further studies using targeted metabolomics and enzyme activity assays are needed to elucidate the underlying mechanisms.

### 4.4 Implications for industrial crop management and essential oil production

From an applied perspective, our results demonstrate that pre-harvest UV irradiation can serve as a precise, low-cost strategy to modulate the volatile composition of tarragon, enhancing its suitability for specific industrial applications. For instance, UV-B treatment could be recommended for producing essential oil with elevated terpenoid content, ideal for traditional perfumery, while combined UV-A+UV-B treatment might be preferred for generating fruity ester-enriched oils for modern fragrance creations. The 12 h daily exposure over 15 days employed in this study is readily implementable in controlled-environment agriculture or greenhouse settings, offering a practical pathway for commercial adoption.

Moreover, the dual-platform volatilomic methodology established here provides a robust framework for quality assessment and breeding selection in aromatic crops. The 122 differential compounds identified (VIP > 1.0) could serve as biomarkers for monitoring UV effects or for screening cultivars with enhanced UV responsiveness. Future research should explore the molecular mechanisms underlying wavelength-specific regulation, including transcriptomic analysis of terpene synthase and alcohol acyltransferase genes, and validate the industrial scalability of these findings through pilot-scale essential oil extraction and olfactory evaluation.

### 4.5 Comparison with previous studies and broader relevance

Our findings are consistent with studies on other aromatic crops, such as sweet basil (Gill and Tuteja, 2010), lemon balm (Bertoli et al., 2013), and thyme (Mumivand et al., 2021), which reported UV-induced modulation of volatile profiles. However, this study is the first to systematically compare UV-A, UV-B, and combined effects on tarragon, revealing that combined treatment can elicit unique responses not predictable from single-wavelength exposures. This highlights the importance of considering spectral interactions in developing UV-based cultivation strategies.

Currently, studies specifically investigating the effects of UV radiation on volatile metabolism in tarragon remain scarce. However, the regulatory mechanisms of UV radiation on plant growth, development, and secondary metabolite accumulation have been extensively documented across diverse plant species, exhibiting notable species conservation and stress-dose specificity. Existing evidence indicates that different UV wavelength bands mediate plant stress adaptation through the regulation of antioxidant systems, metabolic reprogramming, and cell wall remodeling.

UV-A-mediated metabolic regulation. UV-A radiation has been shown to improve fruit color quality in mango by promoting anthocyanin and carotenoid biosynthesis while accelerating chlorophyll degradation, concomitantly enhancing carotenoid accumulation in the pulp (Li et al., 2025). Meanwhile, UV-A exposure induces ROS accumulation, activates cell wall degradation processes, and triggers the antioxidant defense system to counteract UV-induced oxidative damage (Li et al., 2025; J. Wang et al., 2026). Studies on algal systems have further revealed that dose-dependent UV stress reshapes resource allocation and metabolic patterns: transient UV exposure promotes biomass accumulation, moderate UV stress directs nutrients toward photoprotective protein synthesis, while prolonged UV stress shifts metabolic resources toward carbohydrate storage via osmotic regulation, highlighting the remarkable metabolic plasticity of plants in response to UV stress (Ben Ghedifa et al., 2026).

UV-B as a key regulator of secondary metabolism. UV-B radiation plays a critical role in crop quality improvement and stress tolerance regulation. Low-intensity UV-B exposure activates endogenous protective mechanisms, significantly inducing the synthesis of flavonoids and other antioxidant compounds, thereby enhancing ROS scavenging capacity and increasing plant tolerance to UV stress (Shoaib et al., 2023). Studies on highland barley under combined drought and UV-B stress have demonstrated that drought primarily regulates endogenous hormone balance and nutrient metabolism, whereas UV-B further reinforces antioxidant metabolism, resulting in synergistic stress responses (Shoaib et al., 2026). In Brassicaceae edible sprouts, UV-B radiation significantly promotes phenolic secondary metabolite accumulation and enhances overall antioxidant activity, representing a sustainable approach for nutritional quality improvement; although species-specific differences exist, the core regulatory pattern of UV-B-mediated phenolic accumulation is highly conserved across species.

UV-B effects on plant tissue quality and aroma metabolism. UV-B radiation exerts significant remodeling effects on carbohydrate, organic acid, and aroma metabolism. In grape berries, postharvest UV-B treatment significantly increased total soluble solids, sucrose, glucose, and fructose contents, while accelerating organic acid degradation, thereby optimizing the sugar–acid balance and flavor quality (Bian et al., 2026). UV-B also significantly induced the accumulation of polyphenolic compounds including anthocyanins, flavanols, total phenolics, and flavonoids, with specific enrichment effects on individual phenolic monomers (Bian et al., 2026; Santin et al., 2020; X. Wang et al., 2018). At the level of aroma metabolism, UV-B significantly modulates the free and bound fractions of volatile compounds, promoting the specific accumulation of key aroma-contributing aldehydes, ketones, esters, and terpenoids, thereby reshaping the overall volatile profile (Del-Castillo-Alonso et al., 2020; Tan et al., 2025).

Integration with the present findings. The results of the present study on tarragon volatile metabolites are highly consistent with the aforementioned findings. Our data demonstrate that UV-B treatment significantly enhances the accumulation of ketones and terpenoids in tarragon, further corroborating the core function of UV-B radiation in specifically modulating plant aroma metabolic pathways and restructuring volatile profiles. Collectively with previous reports, these findings suggest that UV-B may activate antioxidant secondary metabolic pathways in tarragon, reconfigure carbon allocation patterns, and ultimately drive the specific enrichment of ketone-type aroma compounds. This provides a theoretical basis for the application of UV radiation in modulating the volatile quality of aromatic plants and improving the flavor characteristics of tarragon.

The industrial relevance of this work is underscored by the growing global demand for natural aromatic ingredients, driven by consumer preferences for clean-label products and sustainable sourcing (Kanter et al., 2026; Z. Liu et al., 2026). By demonstrating that UV irradiation can enhance the concentration of high-value compounds, this study offers a natural and environmentally friendly alternative to genetic modification or chemical elicitors for improving essential oil quality.

### 4.6 Methodological considerations and future perspectives

While the present study provides comprehensive volatilomic characterization of tarragon under UV treatments, several methodological limitations should be acknowledged. First, the relative quantification of VOCs was performed using the area normalization method (peak area of individual compound relative to total peak area of all identified volatiles), rather than internal standard-based absolute quantification. Although this approach is widely employed in comparative volatilomics studies for evaluating treatment-induced changes, it is inherently susceptible to variations in total volatile emission across samples. Factors such as differences in sample matrix, extraction efficiency, and instrument response can influence the total peak area, thereby affecting the relative proportions of individual compounds. Consequently, while the observed trends and treatment-specific differences are robust, the reported values should be interpreted as semi-quantitative rather than absolute concentrations.

Second, the HS-GC-IMS platform, despite its high sensitivity and rapid analysis capability, is constrained by database limitations, resulting in 21 unidentified peaks that may represent compounds of sensory or biological significance. The complementary HS-SPME-GC-MS analysis partially mitigated this limitation, but comprehensive identification would benefit from the use of authentic standards and orthogonal analytical techniques.

Third, our study was conducted under laboratory conditions with a single tarragon population; the extent to which these findings can be generalized to other genotypes, cultivation systems, or environmental conditions remains to be determined. Future research should explore genotype-specific responses to UV irradiation and validate the scalability of these findings under field conditions and pilot-scale essential oil extraction.

Fourth, although we observed treatment-specific accumulation of key volatile compounds, the underlying molecular mechanisms—particularly the transcriptional regulation of terpene synthases, alcohol acyltransferases, and other key enzymes in volatile biosynthetic pathways—were not investigated. Integrated transcriptomic and metabolomic analyses would be valuable for elucidating the regulatory networks governing wavelength-specific metabolic reprogramming.

In addition, future studies should incorporate sensory evaluation to assess the organoleptic impact of UV-induced volatile changes, as the industrial value of tarragon essential oil ultimately depends on consumer perception. Finally, the optimization of UV irradiation parameters—including intensity, duration, timing, and spectral quality—warrants systematic investigation to develop practical, cost-effective pre-harvest protocols for commercial adoption.

## 5 Conclusion

This study provides the first comprehensive volatilomic characterization of tarragon (*Artemisia dracunculus* L.) in response to targeted pre-harvest UV irradiation, demonstrating that UV-A, UV-B, and combined UV-A+UV-B treatments elicit distinct metabolic reprogramming events. Through complementary HS-GC-IMS and HS-SPME-GC-MS analyses, 179 volatile compounds were identified, with terpenoids predominating (40–52%) across all treatment groups. OPLS-DA models achieved excellent discrimination among the four experimental groups (*Q*² > 0.95), and VIP analysis resolved 122 wavelength-responsive biomarkers. UV-B irradiation specifically enhanced industrially valuable terpenoids (*γ*-terpinene, *β*-ocimene) and ketones, while combined UV-A+UV-B treatment synergistically increased ester levels, enriching compounds associated with fruity and floral notes desirable in perfumery. Aldehydes were uniformly enhanced across treatments, reflecting UV-induced oxidative stress. These findings establish that spectral quality can be harnessed as a precise pre-harvest tool to tailor tarragon essential oil composition—UV-B for terpenoid-rich profiles suited to classical perfumery, and UV-A+UV-B for ester-enriched oils with enhanced fruity and floral characteristics. The 122 robust biomarkers identified offer a diagnostic toolkit for screening UV-responsive cultivars and quality monitoring. Future research should explore the molecular mechanisms underlying wavelength-specific regulation and validate industrial scalability through pilot-scale essential oil extraction and sensory evaluation.

## Data Availability

The original contributions presented in the study are included in the article/**Supplementary Material**. Further inquiries can be directed to the corresponding author.
