# Supplementary material for: Pre-harvest UV irradiation differentially modulates terpenoids and esters in tarragon (Artemisia dracunculus L.) for enhanced essential oil quality
Source: Front Plant Sci. 2026 Aug 6;17:1883766. doi: 10.3389/fpls.2026.1883766 (PMC13490144; doi:10.3389/fpls.2026.1883766)
Supplement: Supplementary file 1 [file DataSheet1.docx]

**Pre-harvest UV irradiation differentially modulates terpenoids and esters in tarragon (*Artemisia dracunculus* L.) for enhanced essential oil quality**

Chunjing Hou ^a,b^, Haitao Meng ^a,b*^, Jialu Yang ^a,b^, Yiming Sun ^a,b^

^a^ *Xinjiang Key Laboratory of New Energy and Energy Storage Technology, Xinjiang Institute of Technology, Aksu 843100, China*

^b^ *Xinjiang Institute of Technology, Aksu 843100, China*

**Figure S1**. Schematic diagram of the experimental setup and different ultraviolet (UV) treatment regimens.

(a) Experimental apparatus, (b) DZ (ambient UV control, exposed to natural background UV), (c) UV-B-supplemented treatment group, (d) UV-A-supplemented treatment group, (e) Combined UV-A and UV-B supplementation treatment group (UV-A+UV-B).

**Table S1**. HS-GC-IMS identification and quantification of *Artemisia dracunculus* L. under different ultraviolet irradiation conditions

| **Category** | **No** | **Chemicals** | **Cas** | **Formula** | **Molecular weight** | **Retention index（RI）** | **Retention time/s** | **Drift time /ms** | **Relative content（%）** | | | | Odor description |
| --- | --- | --- | --- | --- | --- | --- | --- | --- | --- | --- | --- | --- | --- |
|  |  |  |  |  |  |  |  |  | **DZ** | **UV-A** | **UV-B** | **UV-A+ UV-B** |  |
| **Terpenes** | A1-1 | (R/S)-Limonene -D | C138863 | C_10_H_16_ | 136.2 | 1034.7 | 664.081 | 1.21946 | 2.82±0.10^a^ | 1.54±0.05^c^ | 2.32±0.11^b^ | 2.24±0.04^b^ | lemon, sweet, orange, pine oil |
|  | A1-2 | (R/S)-Limonene -M | C138863 | C_10_H_16_ | 136.2 | 1035 | 664.594 | 1.2918 | 1.72±0.06^ab^ | 1.52±0.01^c^ | 1.77±005^a^ | 1.65±0.07^b^ |  |
|  | A1-3 | (R/S)-Limonene -P-1 | C138863 | C_10_H_16_ | 136.2 | 1033.9 | 662.541 | 1.65347 | 1.31±0.03^a^ | 0.80±0.02^b^ | 0.87±0.13^b^ | 1.27±0.08^a^ |  |
|  | A1-4 | (R/S)-Limonene -P-2 | C138863 | C_10_H_16_ | 136.2 | 1033.9 | 662.541 | 1.72806 | 1.32±0.04^a^ | 0.78±0.02^b^ | 0.85±0.13^b^ | 1.30±0.13^a^ |  |
|  | A2-1 | *α*-Pinene -D | C80568 | C_10_H_16_ | 136.2 | 929.8 | 442.262 | 1.21474 | 1.40±0.02^a^ | 1.09±0.02^d^ | 1.32±0.01^b^ | 1.27±0.02^c^ | Fruity, fresh, camphor, sweet, pine wood |
|  | A2-2 | *α*-Pinene -M | C80568 | C_10_H_16_ | 136.2 | 931 | 444.238 | 1.29312 | 2.59±0.05^b^ | 2.29±0.02^c^ | 2.75±0.01^a^ | 2.58±0.08^b^ |  |
|  | A2-3 | *α*-Pinene -P-1 | C80568 | C_10_H_16_ | 136.2 | 931.6 | 445.225 | 1.66601 | 3.58±0.07^a^ | 2.50±0.06^b^ | 2.62±0.23^b^ | 3.33±0.11^a^ |  |
|  | A2-4 | *α*-Pinene -P-2 | C80568 | C_10_H_16_ | 136.2 | 930.4 | 443.25 | 1.73014 | 1.23±0.03^a^ | 0.89±0.02^b^ | 0.92±0.07^b^ | 1.14±0.08^a^ |  |
|  | A3 | Terpinolene | C586629 | C_10_H_16_ | 136.2 | 1086.2 | 761.424 | 1.22001 | 1.12±0.03^a^ | 0.78±0.01^c^ | 0.52±0.04^d^ | 1.03±0.07^b^ | fresh woody sweet pine citrus |
|  | A4-1 | (*E*)-*β*-ocimene -D | C13877913 | C_10_H_16_ | 136.2 | 1047.9 | 657.079 | 1.71502 | 4.21±0.08^b^ | 3.91±0.05^d^ | 4.44±0.06^a^ | 4.01±0.10^c^ | floral, woody, fruity |
|  | A4-2 | (*E*)-*β*-ocimene -P | C13877913 | C_10_H_16_ | 136.2 | 1046.9 | 655.299 | 2.18081 | 1.45±0.04^b^ | 1.64±0.06^a^ | 1.46±0.08^b^ | 1.50±0.04^b^ |  |
|  | A5-1 | *β*-Pinene -D | C127913 | C_10_H_16_ | 136.2 | 974.1 | 522.766 | 1.21712 | 0.92±0.01^d^ | 1.26±0.03^b^ | 1.81±0.07^a^ | 1.06±0.02^c^ | resin, green |
|  | A5-2 | *β*-Pinene -M | C127913 | C_10_H_16_ | 136.2 | 974.4 | 523.26 | 1.28837 | 3.87±0.06^b^ | 3.39±0.05^c^ | 4.10±0.11^a^ | 3.85±0.18^b^ |  |
|  | A5-3 | *β*-Pinene -P-1 | C127913 | C_10_H_16_ | 136.2 | 974.9 | 524.247 | 1.64701 | 1.91±0.05^d^ | 3.00±0.04^b^ | 3.14±0.01^a^ | 2.66±0.05^c^ |  |
|  | A5-4 | *β*-Pinene -P-2 | C127913 | C_10_H_16_ | 136.2 | 974.6 | 523.754 | 1.72539 | 1.39±0.06^c^ | 2.47±0.05^a^ | 2.40±0.03^a^ | 1.95±0.05^b^ |  |
|  | A5-5 | *β*-Pinene -P-3 | C127913 | C_10_H_16_ | 136.2 | 973.6 | 521.778 | 2.15766 | 1.28±0.04^d^ | 4.89±0.09^a^ | 3.50±0.14^b^ | 2.14±0.21^c^ |  |
|  | A6-1 | *γ*-terpinene -D | C99854 | C_10_H_16_ | 136.2 | 1057.4 | 677.04 | 1.70022 | 5.76±0.05^c^ | 5.36±0.09^d^ | 6.61±0.06^a^ | 6.07±0.20^b^ | oil, wood, terpenes, lemon, lime, herbs |
|  | A6-2 | *γ*-terpinene -P | C99854 | C_10_H_16_ | 136.2 | 1057.1 | 676.426 | 2.16305 | 1.90±0.03^b^ | 1.72±0.02^c^ | 2.20±0.13^a^ | 1.98±0.09^b^ |  |
|  | A7-1 | Myrcene -D | C123353 | C_10_H_16_ | 136.2 | 992 | 559.313 | 1.22129 | 1.98±0.08^a^ | 1.29±0.02^c^ | 1.85±0.04^b^ | 1.81±0.03^b^ | must, spice, balsamic |
|  | A7-2 | Myrcene -M | C123353 | C_10_H_16_ | 136.2 | 992.3 | 559.807 | 1.28336 | 1.80±0.05^b^ | 1.84±0.01^b^ | 2.12±0.06^a^ | 1.83±0.12^b^ |  |
|  | A7-3 | Myrcene -P-1 | C123353 | C_10_H_16_ | 136.2 | 992 | 559.313 | 1.64146 | 0.98±0.01^b^ | 0.60±0.00^d^ | 0.90±0.01^c^ | 1.02±0.01^a^ |  |
|  | A7-4 | Myrcene -P-2 | C123353 | C_10_H_16_ | 136.2 | 991.8 | 558.82 | 1.7107 | 3.52±0.05^a^ | 2.02±0.04^c^ | 3.14±0.11^b^ | 3.59±0.06^a^ |  |
|  | A7-5 | Myrcene -P-3 | C123353 | C_10_H_16_ | 136.2 | 990.6 | 556.35 | 2.16668 | 1.39±0.06^a^ | 0.46±0.05^c^ | 0.88±0.04^b^ | 1.44±0.15^a^ |  |
| **Alcohols** | B1 | (*Z*)-2-Pentenol | C1576950 | C_5_H_10_O | 86.1 | 771.2 | 257.002 | 0.94613 | 0.75±0.01^a^ | 0.74±0.02^a^ | 0.50±0.01^b^ | 0.53±0.02^b^ | green, plastic, rubber |
|  | B2 | 1-Hexanol | C111273 | C_6_H_14_O | 102.2 | 872.9 | 358.906 | 1.32675 | 0.15±0.01^a^ | 0.16±0.00^a^ | 0.08±0.01^a^ | 0.16±0.01^b^ | fresh, fruity, wine, sweet, green |
|  | B3 | 1-Menthol | C2216515 | C_10_H_20_O | 156.3 | 1182.2 | 967.041 | 1.23152 | 0.21±0.04^a^ | 0.06±0.01^bc^ | 0.10±0.01^b^ | 0.05±0.01^c^ | mint |
|  | B4-1 | 1-Pentanol -D | C71410 | C_5_H_12_O | 88.1 | 762.1 | 248.649 | 1.25497 | 0.19±0.02^b^ | 0.26±0.01^a^ | 0.11±0.01^c^ | 0.19±0.02^b^ | balsamic |
|  | B4-2 | 1-Pentanol -M | C71410 | C_5_H_12_O | 88.1 | 764.2 | 250.543 | 1.51269 | 0.52±0.01^a^ | 0.48±0.01^b^ | 0.44±0.02^c^ | 0.51±0.01^a^ |  |
|  | B5 | 2-Methylbutanol | C137326 | C_5_H_12_O | 88.1 | 735.2 | 225.54 | 1.22704 | 0.09±0.00^a^ | 0.07±0.01^b^ | 0.06±0.00^b^ | 0.08±0.00^a^ | roast onion, fruity, floral,wine |
|  | B6-1 | 3-Methylbutanol -D | C123513 | C_5_H_12_O | 88.1 | 731 | 222.131 | 1.24481 | 0.03±0.00^b^ | 0.05±0.00^a^ | 0.03±0.00^b^ | 0.05±0.01^a^ | whiskey, banana, fruity |
|  | B6-2 | 3-Methylbutanol -M | C123513 | C_5_H_12_O | 88.1 | 730.6 | 221.752 | 1.48729 | 0.10±0.01^b^ | 0.10±0.01^b^ | 0.12±0.00^a^ | 0.13±0.00^a^ |  |
|  | B7 | Dihydrolinalool | C78693 | C_10_H_22_O | 158.3 | 1134.9 | 844.029 | 1.26813 | 0.13±0.01^b^ | 0.17±0.00^b^ | 0.21±0.02^a^ | 0.22±0.03^a^ | Floral,Fruity,green, citrus |
|  | B8-1 | 1-Propanethiol -D | C107039 | C_3_H_8_S | 76.2 | 627.8 | 152.522 | 1.17337 | 0.02±0.00^c^ | 0.06±0.00^a^ | 0.04±0.00^b^ | 0.03±0.01^b^ | alliaceous |
|  | B8-2 | 1-Propanethiol -M | C107039 | C_3_H_8_S | 76.2 | 627.1 | 152.208 | 1.3649 | 0.28±0.01^c^ | 0.30±0.01^c^ | 0.40±0.00^a^ | 0.32±0.02^b^ |  |
|  | B9-1 | Epoxydihydrolinalool -D | C1365191 | C_10_H_18_O_2_ | 170.3 | 1100.4 | 765.492 | 1.24181 | 0.05±0.01^c^ | 0.07±0.01^b^ | 0.08±0.00^a^ | 0.07±0.00^b^ | floral |
|  | B9-2 | Epoxydihydrolinalool -M | C1365191 | C_10_H_18_O | 170.3 | 1099 | 762.421 | 1.80585 | 1.04±0.05^c^ | 1.46±0.04^b^ | 1.72±0.13^a^ | 1.43±0.13^b^ |  |
|  | B10 | 1-Penten-3-ol | C616251 | C_5_H_10_O | 86.1 | 685.1 | 180.096 | 0.94448 | 0.53±0.003^a^ | 0.33±0.02^c^ | 0.49±0.02^b^ | 0.46±0.02^b^ | ethereal, green, tropical fruity |
| **Aldehydes** | C1 | 2-Methyl-2-propanal | C78842 | C_4_H_8_O | 72.1 | 564.5 | 128.12 | 1.28147 | 0.38±0.02^c^ | 0.82±0.01^a^ | 0.41±0.01^bc^ | 0.44±0.02^b^ | banana, melon , slightly nutty |
|  | C2-1 | Benzaldehyde -D | C100527 | C_7_H_6_O | 106.1 | 962 | 522.613 | 1.15041 | 0.13±0.02^b^ | 0.34±0.02^a^ | 0.09±0.01^b^ | 0.12±0.03^b^ | bitter almond, cherry, nutty |
|  | C2-2 | Benzaldehyde -M | C100527 | C_7_H_6_O | 106.1 | 960.9 | 520.338 | 1.46748 | 0.63±0.01^b^ | 1.00±0.02^a^ | 0.55±0.04^c^ | 0.55±0.02^c^ |  |
|  | C3-1 | Heptanal -D | C111717 | C_7_H_14_O | 114.2 | 901.1 | 409.712 | 1.33102 | 0.03±0.00^a^ | 0.03±0.00^a^ | 0.03±0.00^a^ | 0.02±0.00^a^ | fresh, aldehyde, fatty, green herbs, wine, fruity |
|  | C3-2 | Heptanal -M | C111717 | C_7_H_14_O | 114.2 | 902.5 | 412.003 | 1.69764 | 0.22±0.01^c^ | 0.25±0.00^b^ | 0.29±0.01^a^ | 0.20±0.00^c^ |  |
|  | C4-1 | 2-Methyl butanal -D | C96173 | C_5_H_10_O | 86.1 | 664.5 | 169.772 | 1.16171 | 1.26±0.03^c^ | 1.63±0.01^a^ | 1.18±0.04^d^ | 1.61±0.07^a^ | Almond, cocoa, malt |
|  | C4-2 | 2-Methyl butanal -M | C96173 | C_5_H_10_O | 86.1 | 663.8 | 169.442 | 1.40099 | 0.41±0.03^b^ | 0.21±0.01^d^ | 0.52±0.01^a^ | 0.35±0.04^c^ |  |
|  | C5-1 | Pentanal -D | C110623 | C_5_H_10_O | 86.1 | 696.2 | 186.94 | 1.1845 | 1.47±0.05^a^ | 1.03±0.04^c^ | 1.14±0.06^b^ | 1.41±0.01^a^ | green grassy, faint banana, pungent |
|  | C5-2 | Pentanal -M | C110623 | C_5_H_10_O | 86.1 | 695.7 | 186.61 | 1.42251 | 0.48±0.02^b^ | 0.30±0.02^a^ | 0.65±0.02^a^ | 0.52±0.05^b^ |  |
|  | C6 | Propanal | C123386 | C_3_H_6_O | 58.1 | 500.2 | 113.143 | 1.05488 | 1.25±0.03^a^ | 0.91±0.07^c^ | 1.25±0.03^a^ | 1.11±0.03^b^ | pungent, green grassy |
|  | C7-1 | 3-Methyl butanal -D | C590863 | C_5_H_10_O | 86.1 | 649.4 | 170.886 | 1.19899 | 0.57±0.02^b^ | 0.75±0.01^a^ | 0.46±0.02^c^ | 0.73±0.03^a^ | chocolate, fat |
|  | C7-2 | 3-Methyl butanal -M | C590863 | C_5_H_10_O | 86.1 | 652.6 | 172.444 | 1.40578 | 0.70±0.03^a^ | 0.46±0.01^b^ | 0.65±0.06^a^ | 0.47±0.00^b^ |  |
|  | C8-1 | 3-Methyl-2-butenal -D | C107868 | C_5_H_8_O | 84.1 | 780.1 | 265.447 | 1.093 | 0.21±0.04^b^ | 0.29±0.01^a^ | 0.32±0.02^a^ | 0.27±0.03^a^ | fruity |
|  | C8-2 | 3-Methyl-2-butenal -M | C107868 | C_5_H_8_O | 84.1 | 779.2 | 264.573 | 1.36186 | 0.42±0.02^c^ | 0.42±0.00^c^ | 0.54±0.01^a^ | 0.48±0.01^b^ |  |
|  | C9-1 | (*E*)-2-Heptenal -D | C18829555 | C_7_H_12_O | 112.2 | 957.5 | 513.24 | 1.25533 | 0.03±0.00^c^ | 0.04±0.00^b^ | 0.06±0.01^a^ | 0.06±0.01^a^ | spicy, green vegetables, fresh, fatty |
|  | C9-2 | (*E*)-2-Heptenal -M | C18829555 | C_7_H_12_O | 112.2 | 956.9 | 512.148 | 1.6675 | 0.31±0.01^c^ | 0.31±0.01^c^ | 0.51±0.02^a^ | 0.45±0.02^b^ |  |
|  | C10-1 | (*E*)-2-Hexenal -D | C6728263 | C_6_H_10_O | 98.1 | 849.5 | 329.071 | 1.18125 | 4.91±0.14^b^ | 7.78±0.09^a^ | 0.61±0.16^d^ | 2.40±0.03^c^ | green, banana, fat |
|  | C10-2 | (*E*)-2-Hexenal -M | C6728263 | C_6_H_10_O | 98.1 | 848 | 327.237 | 1.51455 | 2.33±0.14^b^ | 2.57±0.09^a^ | 1.14±0.14^d^ | 1.64±0.06^c^ |  |
|  | C11-1 | (*E*)-2-Pentenal -D | C1576870 | C_5_H_8_O | 84.1 | 750.9 | 229.53 | 1.10727 | 0.77±0.01^b^ | 1.44±0.05^a^ | 0.46±0.01^c^ | 0.51±0.04^c^ | potato, peas |
|  | C11-2 | (*E*)-2-Pentenal -M | C1576870 | C_5_H_8_O | 84.1 | 749.8 | 228.54 | 1.36174 | 0.66±0.02^a^ | 0.56±0.03^b^ | 0.65±0.01^a^ | 0.59±0.02^b^ |  |
|  | C12 | (*E*)-2.4-Heptadienal | C4313035 | C_7_H_10_O | 110.2 | 1019.4 | 637.642 | 1.19047 | 0.31±0.02^b^ | 0.36±0.01^a^ | 0.20±0.00^d^ | 0.22±0.01^c^ | fatty, oily, aldehyde, vegetable, cinnamon |
|  | C13-1 | 1-Hexanal -D | C66251 | C_6_H_12_O | 100.2 | 791 | 266.508 | 1.25413 | 2.38±0.07^b^ | 2.47±0.06^b^ | 2.86±0.10^a^ | 2.79±0.03^a^ | Fresh, green, fat, fruity |
|  | C13-2 | 1-Hexanal -M | C66251 | C_6_H_12_O | 100.2 | 790 | 265.517 | 1.55671 | 1.02±0.04^b^ | 0.80±0.01^c^ | 1.20±0.05^a^ | 1.05±0.06^b^ |  |
| **Ketones** | D1-1 | 1-Penten-3-one -D | C1629589 | C_5_H_8_O | 84.1 | 686.4 | 180.553 | 1.07963 | 0.55±0.02^b^ | 0.93±0.05^a^ | 0.25±0.01^d^ | 0.40±0.01^c^ | strong pungent odors |
|  | D1-2 | 1-Penten-3-one -M | C1629589 | C_5_H_8_O | 84.1 | 687.2 | 180.957 | 1.31064 | 0.12±0.01^a^ | 0.08±0.00^b^ | 0.12±0.01^a^ | 0.13±0.01^a^ |  |
|  | D2 | 2.3-Butanedione | C431038 | C_4_H_6_O_2_ | 86.1 | 590.1 | 145.171 | 1.18778 | 2.39±0.05^b^ | 2.32±0.02^bc^ | 2.72±0.04^a^ | 2.29±0.03^c^ | butter, popcorn, sweet taste, sour rice |
|  | D3 | 2-Butanone | C78933 | C_4_H_8_O | 72.1 | 597.6 | 140.643 | 1.24799 | 0.50±0.02^a^ | 0.36±0.01^b^ | 0.22±0.03^c^ | 0.33±0.01^b^ | fruity , camphor |
|  | D4-1 | 2-Methyl-2-propenal -D | C78853 | C_4_H_6_O | 70.1 | 576.3 | 139.717 | 1.04924 | 0.49±0.00^c^ | 0.47±0.01^c^ | 0.79±0.03^a^ | 0.61±0.01^b^ | hyacinth foliage |
|  | D4-2 | 2-Methyl-2-propenal -M | C78853 | C_4_H_6_O | 70.1 | 576.8 | 139.911 | 1.22038 | 0.13±0.01^b^ | 0.07±0.00^c^ | 0.20±0.01^a^ | 0.12±0.02^b^ |  |
|  | D5 | 2-Octanone | C111137 | C_8_H_16_O | 128.2 | 1004 | 579.241 | 1.3382 | 0.15±0.04^b^ | 0.10±0.01^c^ | 0.26±0.03^a^ | 0.15±0.01^b^ | mouldy, ketone, milk, cheese,mushroom |
|  | D6-1 | 3-Penten-2-one -D | C625332 | C_5_H_8_O | 84.1 | 736 | 226.135 | 1.07168 | 0.21±0.01^c^ | 0.33±0.02^a^ | 0.28±0.03^b^ | 0.22±0.02^c^ | Fruity, turns into spicy during storage |
|  | D6-2 | 3-Penten-2-one -M | C625332 | C_5_H_8_O | 84.1 | 736 | 226.135 | 1.33817 | 0.18±0.01^b^ | 0.16±0.00^b^ | 0.27±0.02^a^ | 0.18±0.02^b^ |  |
|  | D7 | 6-Methyl. 5 hepten-2-one | C110930 | C_8_H_14_O | 126.2 | 988.5 | 580.944 | 1.17451 | 0.64±0.02^b^ | 0.40±0.00^c^ | 0.56±0.02^b^ | 0.80±0.09^a^ | citrus, fruity, mouldy, ketone |
|  | D8 | Acetone | C67641 | C_3_H_6_O | 58.1 | 533.1 | 117.278 | 1.1136 | 7.60±0.09^a^ | 5.69±0.05^d^ | 7.21±0.13^b^ | 6.89±0.28^c^ | Fresh, apple, pear |
|  | D9 | Cyclohexanone | C108941 | C_6_H_10_O | 98.1 | 899.4 | 406.964 | 1.152 | 0.06±0.00^c^ | 0.11±0.07^a^ | 0.07±0.00^b^ | 0.06±0.00^c^ | strong pungent, earthy |
| **Acids** | E1 | Benzoic acid | C65850 | C_7_H_6_O_2_ | 122.1 | 1202 | 1035.825 | 1.21881 | 0.65±0.11^c^ | 1.40±0.10^a^ | 0.74±0.04^c^ | 1.06±0.06^b^ | Pungent, Sour, faint balsamic |
|  | E2 | Butanoic acid | C107926 | C_4_H_8_O_2_ | 88.1 | 814.6 | 300.17 | 1.16229 | 0.05±0.00^b^ | 0.06±0.00^a^ | 0.03±0.00^c^ | 0.03±0.00^c^ | strong acetic acid, cheese, butter, fruity |
|  | E3 | Acetic acid | C64197 | C_2_H_4_O_2_ | 60.1 | 598.8 | 141.085 | 1.05773 | 0.09±0.01^a^ | 0.04±0.00^c^ | 0.08±0.01^ab^ | 0.07±0.01^b^ | spicy |
|  | E4-1 | Acetoin -D | C513860 | C_4_H_8_O_2_ | 88.1 | 710.2 | 205.951 | 1.05637 | 0.20±0.02^c^ | 0.37±0.02^a^ | 0.23±0.03^bc^ | 0.29±0.08^ab^ | butter, cream |
|  | E4-2 | Acetoin -M | C513860 | C_4_H_8_O_2_ | 88.1 | 709.7 | 205.562 | 1.32734 | 0.38±0.03^c^ | 0.42±0.01^bc^ | 0.53±0.02^a^ | 0.46±0.04^b^ |  |
| **Esters** | F1-1 | Isoamyl acetate -D | C123922 | C_7_H_14_O_2_ | 130.2 | 878.2 | 376.276 | 1.30237 | 0.01±0.00b | 0.02±0.00b | 0.01±0.00b | 0.04±0.01a | sweet, banana, fruity |
|  | F1-2 | Isoamyl acetate -M | C123922 | C_7_H_14_O_2_ | 130.2 | 877.2 | 374.902 | 1.7449 | 0.17±0.01^d^ | 0.24±0.01^b^ | 0.21±0.01^c^ | 0.31±0.01^a^ |  |
|  | F2 | Isobutyl butyrate | C539902 | C_8_H_16_O_2_ | 144.2 | 954 | 506.142 | 1.32605 | 0.13±0.06^a^ | 0.10±0.00^a^ | 0.09±0.03^a^ | 0.10±0.02^a^ | apple,pineapple |
|  | F3 | Methyl acetate | C79209 | C_3_H_6_O_2_ | 74.1 | 547 | 128.807 | 1.19899 | 0.05±0.00^c^ | 0.36±0.01^a^ | 0.07±0.00^b^ | 0.07±0.00^b^ | Ester, Green |
|  | F4 | Prenyl acetate | C1191168 | C_7_H_12_O_2_ | 128.2 | 924.8 | 450.477 | 0.94577 | 0.37±0.00^a^ | 0.28±0.02^b^ | 0.28±0.01^b^ | 0.36±0.00^a^ | fruity |
|  | F5-1 | Propyl butanoate -D | C105668 | C_7_H_14_O_2_ | 130.2 | 896.9 | 402.842 | 1.25941 | 0.17±0.05^a^ | 0.06±0.01^c^ | 0.09±0.03^bc^ | 0.14±0.04^ab^ | pineapple, almond, sweet, banana, fruity |
|  | F5-2 | Propyl butanoate -M | C105668 | C_7_H_14_O_2_ | 130.2 | 896 | 401.468 | 1.68475 | 0.50±0.05^a^ | 0.24±0.01^c^ | 0.36±0.06^b^ | 0.45±0.05^ab^ |  |
|  | F6-1 | Butyl acetate -D | C123864 | C_6_H_12_O_2_ | 116.2 | 808.1 | 293.351 | 1.23973 | 0.05±0.01^b^ | 0.04±0.00^b^ | 0.04±0.01^b^ | 0.11±0.01^a^ | fruity |
|  | F6-2 | Butyl acetate -M | C123864 | C_6_H_12_O_2_ | 116.2 | 808.5 | 293.73 | 1.61679 | 0.33±0.02^b^ | 0.27±0.01^c^ | 0.35±0.04^b^ | 0.45±0.01^a^ |  |
|  | F7-1 | Butyl propanoate -D | C590012 | C_7_H_14_O_2_ | 130.2 | 909.1 | 422.995 | 1.28376 | 0.04±0.04^a^ | 0.01±0.00^a^ | 0.02±0.01^a^ | 0.04±0.01^a^ | earthy, sweet rose |
|  | F7-2 | Butyl propanoate -M | C590012 | C_7_H_14_O_2_ | 130.2 | 908.5 | 422.079 | 1.71769 | 0.27±0.12^a^ | 0.10±0.00^b^ | 0.18±0.06^ab^ | 0.26±0.06^a^ |  |
|  | F8-1 | Acetic acid pentyl ester -D | C628637 | C_7_H_14_O_2_ | 130.2 | 915.5 | 433.988 | 1.31669 | 0.05±0.01^c^ | 0.10±0.00^b^ | 0.03±0.01^d^ | 0.19±0.01^a^ | bananas, apples, pears |
|  | F8-2 | Acetic acid pentyl ester -M | C628637 | C_7_H_14_O_2_ | 130.2 | 915.5 | 433.988 | 1.76065 | 0.37±0.03^b^ | 0.41±0.01^b^ | 0.26±0.04^c^ | 0.63±0.01^a^ |  |
| **Others** | G1 | Allyl sulfide | C592881 | C_6_H_10_S | 114.2 | 862 | 355.206 | 1.12622 | 0.12±0.01^c^ | 0.06±0.00^d^ | 0.28±0.02^a^ | 0.20±0.01^b^ | garlic |
|  | G2 | Diethyl sulfide | C352932 | C_4_H_10_S | 90.2 | 701.5 | 199.523 | 1.04619 | 0.17±0.01^b^ | 0.35±0.02^a^ | 0.16±0.01^b^ | 0.12±0.00^c^ | sulfurous |
|  | G3 | Dimethyl disulfide | C624920 | C_2_H_6_S_2_ | 94.2 | 745.4 | 233.997 | 1.14748 | 0.03±0.00^c^ | 0.14±0.05^a^ | 0.09±0.03^ab^ | 0.05±0.01^bc^ | sulfurous , cabbage, onion |
|  | G4 | Dimethyl sulfide | C75183 | C_2_H_6_S | 62.1 | 544.9 | 121.24 | 0.95967 | 0.25±0.02^c^ | 0.07±0.01^d^ | 0.56±0.06^a^ | 0.43±0.05^b^ | cabbage, sulfur, gasoline |
|  | G5 | 1.4-Cineol | C470677 | C_10_H_18_O | 154.3 | 1015.5 | 630.989 | 1.31827 | 0.09±0.00^b^ | 0.08±0.00^c^ | 0.04±0.00^d^ | 0.11±0.01^a^ | Cool,Camphor, Spice |
|  | G6 | 2-Ethyl-3-methylpyrazine | C15707230 | C_7_H_10_N_2_ | 122.2 | 997.6 | 601.604 | 1.16884 | 0.29±0.00^c^ | 0.36±0.03^b^ | 0.43±0.02^a^ | 0.32±0.02^c^ | nutty, peanut, bready |
| **Unkonws** | H1 | 1 | - | - | - | - | - | - | 0.64±0.02^a^ | 0.65±0.00^a^ | 0.60±0.03^b^ | 0.64±0.02^a^ | - |
|  | H2 | 2 | - | - | - | - | - | - | 0.73±0.02^b^ | 0.64±0.02^c^ | 0.88±0.02^a^ | 0.74±0.02^b^ | - |
|  | H3 | 3 | - | - | - | - | - | - | 0.33±0.03^b^ | 0.38±0.01^a^ | 0.29±0.02^c^ | 0.33±0.02^b^ | - |
|  | H4 | 4 | - | - | - | - | - | - | 0.14±0.02^a^ | 0.04±0.00^c^ | 0.07±0.01^b^ | 0.04±0.00^c^ | - |
|  | H5 | 5 | - | - | - | - | - | - | 1.01±0.01^c^ | 0.95±0.02^d^ | 1.19±0.01^a^ | 1.05±0.01^b^ | - |
|  | H6 | 6 | - | - | - | - | - | - | 0.34±0.02^c^ | 0.46±0.01^a^ | 0.40±0.00^b^ | 0.41±0.02^b^ | - |
|  | H7 | 7 | - | - | - | - | - | - | 0.17±0.00^c^ | 0.11±0.01^d^ | 0.35±0.01^a^ | 0.22±0.01^b^ | - |
|  | H8 | 8 | - | - | - | - | - | - | 0.38±0.04^c^ | 0.36±0.00^c^ | 0.68±0.04^a^ | 0.53±0.06^b^ | - |
|  | H9 | 9 | - | - | - | - | - | - | 0.31±0.00^b^ | 0.30±0.02^b^ | 0.35±0.01^a^ | 0.35±0.01^a^ | - |
|  | H10 | 10 | - | - | - | - | - | - | 0.18±0.02^b^ | 0.37±0.02^a^ | 0.19±0.01^b^ | 0.19±0.02^b^ | - |
|  | H11 | 11 | - | - | - | - | - | - | 0.30±0.03^b^ | 0.29±0.01^c^ | 0.32±0.00^a^ | 0.32±0.00^a^ | - |
|  | H12 | 12 | - | - | - | - | - | - | 0.31±0.01^c^ | 0.52±0.03^a^ | 0.20±0.02^d^ | 0.38±0.02^b^ | - |
|  | H13 | 13 | - | - | - | - | - | - | 0.07±0.00^b^ | 0.08±0.00^a^ | 0.06±0.01^c^ | 0.05±0.00^d^ | - |
|  | H14 | 14 | - | - | - | - | - | - | 0.01±0.00^c^ | 0.06±0.01^a^ | 0.01±0.00^c^ | 0.03±0.00^b^ | - |
|  | H15 | 15 | - | - | - | - | - | - | 0.21±0.02^c^ | 0.13±0.02^d^ | 0.43±0.02^a^ | 0.27±0.01^b^ | - |
|  | H16 | 16 | - | - | - | - | - | - | 0.10±0.00^b^ | 0.14±0.02^a^ | 0.08±0.01^b^ | 0.13±0.01^a^ | - |
|  | H17 | 17 | - | - | - | - | - | - | 0.13±0.01^b^ | 0.14±0.00^a^ | 0.11±0.00^c^ | 0.09±0.01^d^ | - |
|  | H18 | 18 | - | - | - | - | - | - | 0.11±0.01^c^ | 0.21±0.02^bc^ | 0.39±0.13^a^ | 0.27±0.06^ab^ | - |
|  | H19 | 19 | - | - | - | - | - | - | 0.34±0.03^a^ | 0.20±0.03^b^ | 0.18±0.04^b^ | 0.34±0.10^a^ | - |
|  | H20 | 20 | - | - | - | - | - | - | 0.04±0.00^c^ | 0.03±0.00^c^ | 0.06±0.00^a^ | 0.05±0.00^b^ | - |
|  | H21 | 21 | - | - | - | - | - | - | 1.90±0.05^c^ | 2.20±0.03^ab^ | 2.36±0.11^a^ | 2.11±0.15^b^ | - |
| The ion aggregation states M, D, and P refer to monomer, dimer, and polymer, respectively. Different letters within the same row for different ultraviolet irradiation conditions of significant differences at a significance level of *p*<0.05.“—”represents non-detection of the VOCs. Odor description from: https://www.femaflavor.org/flavor-library. | | | | | | | | | | | | | |

**Table S2**. HS-SPME-GC-MS identification and quantification of *Artemisia dracunculus* L. under different ultraviolet irradiation conditions.

| **Category** | **No** | **Chemicals** | **Cas#** | **Formula** | **Molecular** | **Retention index（RI）** | **Retention time（RT）** | **Relative content（%）** | | | | **Odor description** |
| --- | --- | --- | --- | --- | --- | --- | --- | --- | --- | --- | --- | --- |
|  |  |  |  |  | **weight（MW）** |  |  | **DZ** | **UV-A** | **UV-B** | **UV-A+ UV -B** |  |
| **Terpenoids** | A1 | *α*-Pinene | 80-56-8 | C_10_H_16_ | 136.1 | 1021 | 6.979 | 1.31±0.04^a^ | 0.09±0.03^c^ | 0.37±0.01^b^ | 0.37±0.03^b^ | Fruity, fresh, camphor, sweet, pine wood |
|  | A2 | Camphene | 79-92-5 | C_10_H_16_ | 136.1 | 1063 | 8.318 | 0.04±0.01^a^ | — | — | — | — |
|  | A3 | *β*-Pinene | 18172-67-3 | C_10_H_16_ | 136.1 | 1105 | 9.662 | 0.62±0.04^a^ | 0.05±0.02^c^ | 0.50±0.07^b^ | 0.12±0.03^c^ | resin, green |
|  | A4 | *α*-Phellandrene | 99-83-2 | C_10_H_16_ | 136.1 | 1121 | 10.169 | 0.10±0.02^a^ | 0.01±0.01^b^ | 0.03±0.02^b^ | 0.04±0.01^b^ | — |
|  | A5 | (*E*)-*β*-ocimene | 13877-91-3 | C_10_H_16_ | 136.2 | 1252 | 14.471 | 24.32±0.03^b^ | 38.76±0.31^a^ | 17.25±0.06^c^ | 14.29±0.10^d^ | floral, woody, fruity |
|  | A6 | Myrcene | 123-35-3 | C_10_H_16_ | 136.1 | 1166 | 11.639 | 3.14±0.02^a^ | 1.14±0.12^d^ | 1.73±0.17^b^ | 1.40±0.10^c^ | must, spice, balsamic |
|  | A7 | *α*-Terpinene | 99-86-5 | C_10_H_16_ | 136.1 | 1177 | 11.986 | — | — | 0.05±0.02^a^ | — | — |
|  | A8 | Limonene | 138-86-3 | C_10_H_16_ | 136.2 | 1200 | 12.719 | 5.24±0.06^a^ | 0.26±0.10^d^ | 1.36±0.04^c^ | 1.56±0.13^b^ | lemon, sweet, orange, pine oil |
|  | A9 | *β*-Phellandrene | 555-10-2 | C_10_H_16_ | 136.1 | 1206 | 12.895 | — | — | 0.03±0.05^a^ | — | — |
|  | A10 | 1,3,8-p-Menthatriene | 18368-95-1 | C_10_H_16_ | 134.1 | 1457 | 19.902 | 1.32±0.03^a^ | 1.07±0.09^c^ | 1.17±0.08^ab^ | 1.27±0.10^a^ | — |
|  | A11 | *trans*-*β*-Ocimene | 3779-61-1 | C_10_H_16_ | 136.1 | 1240 | 13.948 | 34.25±0.05^b^ | 36.59±0.13^a^ | 28.52±0.19^c^ | 23.98±0.13^d^ | floral, woody, fruity |
|  | A12 | *γ*-terpinene | 99-85-5 | C_10_H_16_ | 136.1 | 1244 | 14.212 | 0.03±0.01^a^ | 0.01±0.01^b^ | 0.03±0.02^a^ | — | oil, wood, terpenes, lemon, lime, herbs |
|  | A13 | 3-Carene | 498-15-7 | C_10_H_16_ | 136.1 | 1269 | 14.818 | — | 0.06±0.02^a^ | — | — | — |
|  | A14 | Terpinolene | 586-62-9 | C_10_H_16_ | 136.1 | 1280 | 15.264 | 0.03±0.01^c^ | 0.93±0.04^b^ | 5.93±0.08^a^ | 0.08±0.02^c^ | fresh woody sweet pine citrus |
|  | A15 | 1,3,5,7-Cyclooctatetraene | 629-20-9 | C_8_H_8_ | 104.1 | 1266 | 14.746 | 0.26±0.04^a^ | 0.05±0.03^b^ | — | — | — |
|  | A16 | (*E*)-4,8-Dimethylnona-1,3,7-triene | 19945-61-0 | C_11_H_18_ | 150.1 | 1308 | 16.008 | — | 0.01±0.01^a^ | 0.01±0.01^a^ | — | — |
|  | A17 | Pseudolimonene | 499-97-8 | C_10_H_16_ | 136.1 | 1323 | 16.4153 | — | — | 0.06±0.01^a^ | — | — |
|  | A18 | Pentamethylcyclopentadiene | 4045-44-7 | C_10_H_16_ | 136.1 | 1402 | 18.607 | 0.46±0.03^b^ | — | 0.55±0.06^a^ | 0.33±0.04^c^ | — |
|  | A19 | 2,4,6-Octatriene, 2,6-dimethyl-, (*E,Z*)- | 7216-56-0 | C_10_H_16_ | 136.1 | 1408 | 18.762 | 1.20±0.03^b^ | 1.69±0.02^a^ | 1.22±0.06^b^ | 1.20±0.06^b^ | — |
|  | A20 | Benzene, 1-ethenyl-3,5-dimethyl- | 5379-20-4 | C_10_H_12_ | 132.1 | 1448 | 19.699 | 0.16±0.03^b^ | 0.15±0.03^b^ | 0.45±0.06^a^ | 0.09±0.01^b^ | — |
|  | A21 | *δ*-elemene | 20307-84-0 | C_15_H_24_ | 204.2 | 1474 | 20.282 | 0.20±0.03^b^ | 0.07±0.04^c^ | 0.79±0.08^a^ | 0.76±0.10^a^ | — |
|  | A22 | *γ*-elemene | 29873-99-2 | C_15_H_24_ | 204.2 | 1484 | 20.514 | 0.18±0.04^a^ | 0.08±0.03^c^ | 0.17±0.03^a^ | 0.14±0.05^ab^ | — |
|  | A23 | *α*-Copaene | 3856-25-5 | C_15_H_24_ | 204.2 | 1496 | 20.789 | 0.19±0.04^ab^ | 0.12±0.03^bc^ | 0.27±0.08^a^ | 0.07±0.04^c^ | — |
|  | A24 | p-Mentha-1,5,8-triene | 3642-14-6 | C_15_H_24_ | 134.1 | 1435 | 19.379 | — | 0.01±0.01^b^ | 0.04±0.02^a^ | — | — |
|  | A25 | *α*-Farnesene | 502-61-4 | C_15_H_24_ | 204.2 | 1515 | 21.16 | 0.04±0.02^a^ | — | — | — | — |
|  | A26 | *α*-cedrene | 50894-66-1 | C_15_H_24_ | 204.2 | 1518 | 21.213 | — | 0.01±0.01^b^ | 0.04±0.03^a^ | — | — |
|  | A27 | (-)-*β*-Bourbonene | 5208-59-3 | C_15_H_24_ | 204.2 | 1525 | 21.346 | — | 0.03±0.02^b^ | 0.34±0.05^a^ | — | — |
|  | A28 | (-)-*α*-gurjunene | 489-40-7 | C_15_H_24_ | 204.2 | 1535 | 21.527 | 0.09±0.05^a^ | 0.11±0.04^a^ | 0.16±0.02^a^ | 0.11±0.06^a^ | — |
|  | A29 | Cubebene | 17699-14-8 | C_15_H_24_ | 204.2 | 1797 | 25.615 | 0.18±0.02^ab^ | 0.1±0.02^b^ | 0.27±0.11^a^ | 0.11±0.01^b^ | — |
|  | A30 | (-)-alpha-cedrene | 22567-43-7 | C_15_H_24_ | 204.2 | 1570 | 22.332 | 0.09±0.01^d^ | 0.43±0.05^c^ | 1.28±0.03^a^ | 0.56±0.02^b^ | — |
|  | A31 | *trans*-*α*-Bergamotene | 17699-05-7 | C_15_H_24_ | 204.2 | 1578 | 22.502 | — | — | — | 0.07±0.01^a^ | — |
|  | A32 | Aristolene | 26783-22-2 | C_15_H_24_ | 204.2 | 1585 | 22.469 | 0.07±0.02^a^ | — | — | — | — |
|  | A33 | *β*-Elemene | 515-13-9 | C_15_H_24_ | 204.2 | 1596 | 22.657 | 0.07±0.03^b^ | 0.04±0.03^b^ | 0.15±0.05^a^ | 0.15±0.04^a^ | — |
|  | A34 | *β*-Patchoulene | 514-51-2 | C_15_H_24_ | 204.2 | 1602 | 22.772 | 0.07±0.02^d^ | 0.39±0.05^c^ | 1.10±0.01^a^ | 0.45±0.05^b^ | — |
|  | A35 | *β*-Caryophyllene | 87-44-5 | C_15_H_24_ | 204.2 | 1606 | 22.827 | 0.55±0.04^d^ | 2.45±0.04^a^ | 1.91±0.09^b^ | 0.72±0.10^c^ | — |
|  | A36 | B-Cedrene | 546-28-1 | C_15_H_24_ | 204.2 | 1609 | 22.882 | — | 0.53±0.06^c^ | 1.57±0.15^a^ | 0.85±0.04^b^ | — |
|  | A37 | Aromandendrene | 489-39-4 | C_15_H_24_ | 204.2 | 1617 | 23.004 | 0.34±0.02^c^ | 0.41±0.05^bc^ | 0.67±0.13^a^ | 0.54±0.07^ab^ | — |
|  | A38 | *α*-Guaiene | 52026-55-8 | C_15_H_24_ | 204.2 | 1625 | 23.13 | 0.09±0.01^a^ | 0.06±0.02^a^ | 0.10±0.05^a^ | 0.07±0.03^a^ | — |
|  | A39 | isoledene | 95910-36-4 | C_15_H_24_ | 204.2 | 1643 | 23.406 | 0.03±0.02^a^ | 0.01±0.01^a^ | 0.03±0.02^a^ | 0.03±0.02^a^ | — |
|  | A40 | Bicyclosesquiphellandrene | 7216-56-0 | C_10_H_16_ | 136.1 | 1649 | 23.499 | 0.06±0.02^a^ | 0.04±0.02^a^ | — | — | — |
|  | A41 | 1,5-Cycloundecadiene, 8,8-dimethyl-9-methylene- | 62338-54-9 | C_14_H_22_ | 190.2 | 1652 | 23.544 | 0.05±0.03^a^ | 0.02±0.01^a^ | 0.06±0.02^a^ | 0.03±0.02^a^ | — |
|  | A42 | Alloaromadendrene | 25246-27-9 | C_15_H_24_ | 204.2 | 1658 | 23.632 | 0.19±0.02^a^ | 0.11±0.07^b^ | 0.27±0.04^a^ | 0.25±0.03^a^ | — |
|  | A43 | (+)-Prezizaene | 18444-94-5 | C_15_H_24_ | 204.2 | 1663 | 23.72 | — | 0.01±0.01^c^ | 0.33±0.04^a^ | 0.16±0.04^b^ | — |
|  | A44 | (*E*)-*β*-Farnesene | 18794-84-8 | C_15_H_24_ | 204.2 | 1667 | 23.775 | 0.99±0.01^b^ | 0.31±0.02^c^ | 2.82±0.07^a^ | 1.03±0.07^b^ | — |
|  | A45 | (+)-Ledene | 21747-46-6 | C_15_H_24_ | 204.2 | 1707 | 24.386 | 0.21±0.04^a^ | 0.25±0.07^a^ | 0.24±0.06^a^ | 0.25±0.05^a^ | — |
|  | A46 | *γ*-Muurolene | 30021-74-0 | C_15_H_24_ | 204.2 | 1697 | 24.243 | 0.27±0.07^a^ | 0.20±0.07^a^ | — | — | — |
|  | A47 | *β*-Cadinene） | 5951-61-1 | C_15_H_24_ | 204.2 | 1700 | 24.293 | — | 0.09±0.07^a^ | 0.09±0.01^a^ | — | — |
|  | A48 | *α*-Humulene | 6753-98-6 | C_15_H_24_ | 204.2 | 1683 | 24.028 | — | 0.27±0.03^b^ | 0.41±0.07^a^ | — | — |
|  | A49 | *α*-*cis*-Bergamotene | 18252-46-5 | C_15_H_24_ | 204.2 | 1725 | 24.634 | — | 2.92±0.11^a^ | — | 2.32±0.13^b^ | — |
|  | A50 | Germacrene D | 23986-74-5 | C_15_H_24_ | 204.2 | 1724 | 24.623 | 2.74±0.14^b^ | — | 4.30±0.27^a^ | — | — |
|  | A51 | *α*-amorphene | 20085-19-2 | C_15_H_24_ | 204.2 | 1735 | 24.772 | 0.12±0.03^b^ | 0.23±0.07^a^ | — | — | — |
|  | A52 | bicyclogermacrene | 24703-35-3 | C_15_H_24_ | 204.2 | 1749 | 24.959 | 2.8±0.04^c^ | 2.23±0.17^d^ | 3.39±0.09^a^ | 3.07±0.05^b^ | — |
|  | A53 | *β*-Cadinene | 523-47-7 | C_15_H_24_ | 204.2 | 1767 | 25.196 | 0.10±0.02^ab^ | 0.14±0.04^b^ | 0.23±0.04^a^ | 0.06±0.01^c^ | — |
|  | A54 | *β*-Copaene | 18252-44-3 | C_15_H_24_ | 204.2 | 1773 | 25.279 | 0.16±0.02^a^ | 0.18±0.01^a^ | — | — | — |
|  | A55 | Hotrienol | 20053-88-7 | C_10_H_16_O | 170.1 | 1772 | 25.273 | — | — | 0.63±0.10^a^ | 0.27±0.09^b^ | — |
|  | A56 | *α*-Bisabolene | 29837-07-8 | C_15_H_24_ | 204.2 | 1779 | 25.361 | — | 0.16±0.02^b^ | 0.26±0.05^a^ | — | — |
|  | A57 | *β*-sesquiphellandrene | 20307-83-9 | C_15_H_24_ | 204.2 | 1783 | 25.361 | 0.02±0.01^b^ | — | — | 2.52±0.11^a^ | — |
|  | A58 | *α*-Curcumene | 644-30-4 | C_15_H_22_ | 202.2 | 1781 | 25.394 | — | — | 0.11±0.05^a^ | — | — |
|  | A59 | Dehydrocycloisolongifolen | 74842-33-4 | C_15_H_22_ | 202.2 | 1789 | 25.504 | — | — | — | 0.06±0.02^a^ | — |
|  | A60 | (+)-Limonene oxide | 1195-92-2 | C_10_H_16_O | 152.1 | 1804 | 25.692 | 0.18±0.02^b^ | 0.18±0.07^b^ | — | 0.34±0.01^a^ | — |
|  | A61 | (+)-4*α*-hydroxy-2-carene | 206115-88-0 | C_10_H_16_O | 152.1 | 1823 | 25.929 | 0.24±0.02^b^ | 0.22±0.06^b^ | 0.22±0.05^b^ | 0.41±0.04^a^ | — |
|  | A62 | *β*-Maaliene | 489-29-2 | C_15_H_24_ | 204.2 | 1842 | 26.155 | — | 0.02±0.01^a^ | — | — | — |
|  | A63 | Calamenene | 72937-55-4 | C_15_H_22_ | 202.2 | 1851 | 26.27 | 0.13±0.08^a^ | 0.06±0.02^ab^ | 0.07±0.2^ab^ | — | — |
|  | A64 | *α*-Calacorene | 50277-34-4 | C_15_H_20_ | 200.2 | 1942 | 27.328 | 0.02±0.01^a^ | 0.01±0.01^a^ | 0.04±0.02^a^ | 0.03±0.02^a^ | — |
|  | A65 | o-Cymene | 527-84-4 | C_10_H_14_ | 134.1 | 1270 | 14.967 | 0.02±0.01^b^ | 0.03±0.02^b^ | 0.17±0.02^a^ | 0.01±0.01^b^ | — |
|  | A66 | Guaiazulene | 489-84-9 | C_15_H_18_ | 198.1 | 2459 | 33.266 | 0.05±0.03^a^ | 0.01±0.01^a^ | 0.03±0.02^a^ | 0.03±0.02^a^ | — |
|  | A67 | (*E*)-*γ*-Bisabolene | 53585-13-0 | C_15_H_24_ | 204.2 | 1896 | 26.816 | 0.17±0.03^bc^ | 0.21±0.02^ab^ | 0.28±0.07^a^ | 0.12±0.04^c^ | — |
|  | A68 | 1,4-Dimethylazulene | 1127-69-1 | C_12_H_12_ | 156.1 | 1969 | 27.62 | — | — | — | 0.01±0.01^a^ | — |
| **Ketones** | B1 | L(-)-Carvone | 6485-40-1 | C_10_H_14_O | 150.1 | 1760 | 25.1 | 0.02±0.04^a^ | — | — | 0.02±0.01^a^ | — |
|  | B2 | Acetone | 67-64-1 | C_3_H_6_O | 58 | 831 | 2.66 | 0.17±0.02^b^ | 0.17±0.03^b^ | 0.28±0.04^a^ | 0.19±0.01^b^ | Fresh, apple, pear |
|  | B3 | Methyl vinyl ketone | 78-94-4 | C_4_H_6_O | 70 | 960 | 5.27 | 0.03±0.02^a^ | 0.04±0.02^a^ | 0.02±0.01^a^ | 0.03±0.01^a^ | — |
|  | B4 | 5-Hepten-2-one, 6-methyl- | 110-93-0 | C_8_H_14_O | 126.1 | 1342 | 17 | 0.01±0.01^a^ | 0.01±0.03^a^ | 0.03±0.02^a^ | 0.01±0.05^a^ | — |
|  | B5 | 1,5-Heptadien-4-one, 3,3,6-trimethyl- | 546-49-6 | C_10_H_16_O | 152.1 | 1521 | 21.36 | 0.1±0.01^a^ | — | — | 0.03±0.02^b^ | — |
|  | B6 | 2-Methyl-6-methyleneocta-2,7-dien-4-one | 539-70-8 | C_10_H_14_O | 150.1 | 1422 | 19.09 | 0.08±0.02^a^ | 0.01±0.01^d^ | 0.06±0.02^ab^ | 0.03±0.04^b^ | — |
|  | B7 | (*Z*)-Cinerone | 17190-71-5 | C_10_H_14_O | 150.1 | 1547 | 22.08 | 0.1±0.02^a^ | 0.07±0.01^a^ | 0.11±0.06^a^ | 0.12±0.03^a^ | — |
|  | B8 | *trans*,*trans*-3,5-Heptadien-2-one | 18402-90-9 | C_7_H_10_O | 110.1 | 1565 | 22.18 | 0.07±0.03a | 0.05±0.04^a^ | 0.1±0.02^a^ | — | — |
|  | B9 | *γ*-himachalene | 53111-25-4 | C_15_H_2_4 | 204.2 | 1735 | 24.77 | — | — | 0.34±0.01^a^ | 0.24±0.02^b^ | — |
|  | B10 | *β*-ionone | 14901-07-6 | C_13_H_20_O | 192.2 | 1964 | 27.57 | 0.05±0.03^a^ | 0.03±0.02^a^ | 0.03±0.02^a^ | 0.02±0.01^a^ | — |
|  | B11 | 4-(2,2-Dimethyl-6-methylenecyclohexylidene)-3- | 93175-74-7 | C_14_H_22_O | 206.2 | 1976 | 27.7 | — | — | — | 0.05±0.01^a^ | — |
|  |  | methylbutan-2-one |  |  |  |  |  |  |  |  |  |  |
|  | B12 | Benzophenone | 119-61-9 | C_13_H_10_O | 210.1 | 2535 | 34.51 | — | 0.01±0.01^a^ | — | — | — |
|  | B13 | Capillin | 495-74-9 | C_12_H_8_O | 140.1 | 2042 | 28.4 | — | — | — | 0.19±0.04^a^ | — |
|  | B14 | Geranylacetone | 125284-20-0 | C_14_H_22_O_3_ | 238.2 | 2068 | 28.67 | — | 0.01±0.03^a^ | — | — | — |
|  | B15 | 1H-Pyrrole-2,5-dione, 3-ethyl-4-methyl- | 20189-42-8 | C_7_H_9_NO_2_ | 139.1 | 2298 | 31.11 | 0.04±0.02^ab^ | 0.03±0.02^ab^ | 0.06±0.02^a^ | 0.02±0.01^b^ | — |
| **Esters** | C1 | Diethyl phthalate | 84-66-2 | C_12_H_14_O_4_ | 222.1 | 2403 | 32.43 | 0.08±0.02^b^ | 0.05±0.03^b^ | 0.08±0.02^b^ | 0.13±0.04^a^ | — |
|  | C2 | Dihydroactinidiolide | 17092-92-1 | C_11_H_16_O_2_ | 180.1 | 2423 | 32.73 | 0.09±0.01^a^ | 0.05±0.03^a^ | 0.08±0.02^a^ | 0.06±0.02^a^ | — |
|  | C3 | Diisobutyl phthalate | 84-69-5 | C_16_H_22_O_4_ | 278.2 | 2564 | 35.04 | 0.04±0.03^a^ | 0.02±0.02^ab^ | 0.02±0.01^ab^ | — | — |
|  | C4 | Octaethylene glycol monododecyl ether | 3055-98-9 | C_28_H_58_O_9_ | 538.4 | 2134 | 29.36 | — | 0.06±0.02^a^ | 0.01±0.01^b^ | — | — |
|  | C5 | Dibutyl phthalate | 84-74-2 | C_16_H_22_O_4_ | 278.2 | 2627 | 38.4 | — | 0.02±0.02^ab^ | 0.04±0.03^a^ | — | — |
|  | C6 | Propanoic acid, butyl ester | 590-01-2 | C_7_H_14_O_2_ | 130.1 | 1145 | 10.97 | — | 0.01±0.01^a^ | — | — | — |
|  | C7 | Ethyl Acetate | 141-78-6 | C_4_H_8_O_2_ | 88.1 | 902 | 3.78 | 0.02±0.02^ab^ | 0.03±0.02^a^ | 0.01±0.01^ab^ | — | — |
|  | C8 | 2-Propenoic acid, butyl ester | 141-32-2 | C_7_H_12_O_2_ | 128.1 | 1181 | 12.12 | — | 0.04±0.03^a^ | — | — | — |
|  | C9 | Butanoic acid, butyl ester | 109-21-7 | C_8_H_16_O_2_ | 144.1 | 1222 | 13.4 | — | 0.02±0.02^a^ | — | — | — |
|  | C10 | Cyclopentanecarboxylic acid, ethenyl ester | 16523-06-1 | C_8_H_12_O_2_ | 140.1 | 1438 | 19.46 | 0.09±0.03^a^ | 0.05±0.01^a^ | 0.07±0.03^a^ | 0.06±0.04^a^ | — |
|  | C11 | *cis*-3-Hexenyl isovalerate | 35154-45-1 | C_11_H_20_O_2_ | 184.1 | 1489 | 20.64 | — | — | 0.09±002^a^ | — | — |
|  | C12 | Methyl dihydrojasmonate | 166022-17-9 | C_12_H_20_O_2_S | 228.1 | 1986 | 27.81 | — | — | — | 0.01±0.01^a^ | — |
|  | C13 | Methyl Cinnamate | 1754-62-7 | C_10_H_10_O_2_ | 162.1 | 1983 | 27.78 | — | — | 0.02±0.01^a^ | — | — |
|  | C14 | Ethyl (*E*)-cinnamate | 4610-69-9 | C_11_H_12_O_2_ | 176.1 | 2169 | 29.71 | 0.07±0.04^a^ | — | — | — | — |
|  | C15 | gamma-Decanolactone | 706-14-9 | C_10_H_18_O_2_ | 170.1 | 2188 | 29.9 | 0.13±0.06^a^ | 0.13±0.04^a^ | — | — | — |
|  | C16 | Methyl (*E*)-cinnamate | 1754-62-7 | C_10_H_10_O_2_ | 162.1 | 2120 | 29.21 | 0.06±0.02^a^ | 0.05±0.03^a^ | 0.10±0.06^a^ | 0.04±0.03^a^ | — |
|  | C17 | Ethyl Cinnamate | 4610-69-9 | C_11_H_12_O_2_ | 176.1 | 2167 | 29.69 | — | 0.03±0.03^a^ | — | — | — |
|  | C18 | Borneol acetate | 20347-65-3 | C_12_H_20_O_2_ | 196.1 | 1588 | 22.51 | 0.42±0.07^a^ | 0.02±0.02^b^ | 0.06±0.04^c^ | — | — |
|  | C19 | *γ*-Decalactone | 706-14-9 | C_10_H_18_O_2_ | 170.1 | 2187 | 29.89 | — | 0.06±0.02^a^ | — | — | — |
| **Alcohols** | D1 | Ethanol | 64-17-5 | C_2_H_6_O | 46 | 945 | 4.89 | 0.02±0.02^a^ | 0.02±0.02^a^ | 0.02±0.01^a^ | 0.01±0.01^a^ | — |
|  | D2 | 1,5-Hexadien-3-ol | 924-41-4 | C_6_H_10_O | 98.1 | 1160 | 11.45 | — | — | 0.02±0.02^a^ | — | — |
|  | D3 | 2-Phenyl-1,3-propanediol | 1570-95-2 | C_9_H_12_O_2_ | 152.1 | 1264 | 14.69 | — | — | 0.11±0.03^a^ | — | — |
|  | D4 | 2-Methylbenzyl Alcohol | 89-95-2 | C_8_H_10_O | 122.1 | 1265 | 14.71 | — | — | — | 0.09±0.01^a^ | — |
|  | D5 | 2-Octanol | 123-96-6 | C_8_H_18_O | 130.1 | 1419 | 19.02 | 0.02±0.02^b^ | 0.02±0.01^b^ | 0.10±0.02^a^ | 0.02±0.02^b^ | — |
|  | D6 | 10-Undecyn-1-ol | 2774-84-7 | C_11_H_20_O | 168.2 | 1423 | 19.12 | — | — | — | 0.02±0.01^a^ | — |
|  | D7 | *trans*,*cis*-2,6-Nonadien-1-ol | 28069-72-9 | C_8_H_14_O | 126.1 | 1428 | 19.22 | 0.03±0.02^a^ | — | — | — | — |
|  | D8 | 1-Octen-3-ol | 3391-86-4 | C_8_H_16_O | 128.1 | 1449 | 19.72 | — | — | 0.36±0.03^a^ | 0.08±0.03^b^ | — |
|  | D9 | 1-Hexanol, 2-ethyl- | 104-76-7 | C_8_H_18_O | 130.1 | 1488 | 20.61 | 0.14±0.05^ab^ | 0.09±0.01^bc^ | 0.18±0.04^a^ | 0.06±0.03^c^ | — |
|  | D10 | Citronellol | 106-22-9 | C_10_H_20_O | 156.2 | 1505 | 20.98 | 0.04±0.02^a^ | — | — | 0.02±0.02^ab^ | — |
|  | D11 | Linalool | 78-70-6 | C_10_H_18_O | 154.1 | 1544 | 21.76 | — | 0.19±0.03^b^ | 0.31±0.06^a^ | 0.25±0.08^ab^ | — |
|  | D12 | (-)-*trans*-pinocarveol | 547-61-5 | C_10_H_16_O | 152.1 | 1716 | 24.51 | 0.32±0.06^a^ | 0.07±0.07^b^ | 0.34±0.10^a^ | 0.33±0.04^a^ | — |
|  | D13 | T-Cadinol | 6813-21-4 | C_15_H_24_ | 204.2 | 1675 | 23.9 | — | — | 0.01±0.01^a^ | — | — |
|  | D14 | (*Z*)-carveol | 99-48-9 | C_10_H_16_O | 152.1 | 1876 | 26.57 | 0.06±0.02^ab^ | 0.04±0.03^b^ | 0.09±0.03^a^ | 0.07±0.03^ab^ | — |
|  | D15 | Coniferyl alcohol | 129350-09-0 | C_20_H_28_O_3_ | 316.2 | 2194 | 29.96 | — | 0.01±0.01^a^ | — | — | — |
|  | D16 | Spathulenol | 77171-55-2 | C_15_H_24_O | 220.2 | 2279 | 30.9 | 0.46±0.06^c^ | 0.43±0.02^c^ | 0.73±0.11^b^ | 0.98±0.12^a^ | — |
|  | D17 | 2-Butanone, 3-methyl-1-phenyl- | 2893/5/2 | C_11_H_14_O | 162.1 | 1046 | 7.78 | 0.17±0.05^a^ | 0.06±0.02^b^ | 0.10±0.05^ab^ | 0.08±0.03^b^ | — |
|  | D18 | Isospathulenol | 88395-46-4 | C_15_H_24_O | 220.2 | 2250 | 30.58 | 0.09±0.08^b^ | 0.03±0.02^b^ | 0.08±0.02^b^ | 0.24±0.03^a^ | — |
| **Aldehydes** | E1 | Acetaldehyde | 75-07-0 | C_2_H_4_O | 44 | 725 | 1.58 | — | — | 0.02±0.01^a^ | — | — |
|  | E2 | Propanal | 123-38-6 | C_3_H_6_O | 58 | 812 | 2.35 | — | — | 0.02±0.02^a^ | — | pungent, green grassy |
|  | E3 | Propanal, 2-methyl- | 78-84-2 | C_4_H_8_O | 72.1 | 825 | 2.57 | 0.02±0.01^a^ | 0.01±0.01^a^ | 0.02±0.01^a^ | 0.01±0.01^a^ | — |
|  | E4 | Hexanal | 66-25-1 | C_6_H_12_O | 100.1 | 1095 | 9.34 | 0.05±0.03^a^ | — | — | — | — |
|  | E5 | 2-Hexenal, (*E*)- | 6728-26-3 | C_6_H_10_O | 98.1 | 1231 | 13.66 | 0.01±0.01^a^ | 0.01±0.01^a^ | 0.03±0.02^a^ | 0.01±0.01^a^ | — |
|  | E6 | Citral | 5392-40-5 | C_10_H_16_O | 152.1 | 1372 | 17.83 | 0.24±0.04^a^ | 0.25±0.04^a^ | 0.17±0.03^a^ | 0.20±0.05^a^ | — |
|  | E7 | Decanal | 112-31-2 | C_10_H_20_O | 156.2 | 1505 | 20.98 | — | — | 0.04±0.02^a^ | — | — |
|  | E8 | beta-Cyclocitral | 432-25-7 | C_10_H_16_O | 152.1 | 1636 | 23.3 | — | 0.02±0.02^a^ | — | — | — |
|  | E9 | 2,6-Nonadienal, 3,7-dimethyl | 41448-29-7 | C_11_H_18_O | 166.1 | 1639 | 23.35 | 0.05±0.01^a^ | — | — | — | — |
|  | E10 | Piperidine-1-carbaldehyde | 2591-86-8 | C_6_H_11_NO | 113.1 | 1813 | 25.81 | 0.13±0.04^b^ | 0.15±0.03^b^ | 0.35±0.03^a^ | 0.12±0.03^b^ | — |
|  | E11 | Citronellal | 1000185-00-9 | C_10_H_16_O_2_ | 168.1 | 1955 | 27.47 | — | — | 0.07±0.02^a^ | — | — |
|  | E12 | 2,3-expoxycitral | 16996-12-6 | C_16_H_24_O_5_ | 296.2 | 1998 | 27.95 | — | — | — | 0.01±0.01^a^ | — |
| **Acids** | F1 | Hexanoic acid | 1000186-04-7 | C_9_H_13_NO | 151.1 | 1838 | 26.11 | — | — | 0.01±0.03^a^ | — | — |
|  | F2 | 1-Hexanoic acid | 142-62-1 | C_6_H_12_O_2_ | 116.1 | 1846 | 26.21 | — | — | 0.30±0.02^a^ | — | — |
|  | F3 | Pterin-6-carboxylic acid | 948-60-7 | C_7_H_5_N_5_O_3_ | 207 | 1956 | 27.48 | 0.01±0.01^a^ | — | — | — | — |
|  | F4 | Hexanoic acid, 6-bromo-6 | 4224-70-8 | C_6_H_11_BrO_2_ | 194 | 1631 | 23.22 | — | — | 0.01±0.01^a^ | 0.01±0.01^a^ | — |
|  | F5 | Hexanoic acid, 2-methyl- | 4536-23-6 | C_7_H_14_O_2_ | 130.1 | 1671 | 23.85 | 0.11±0.01^a^ | 0.03±0.02^b^ | 0.08±0.02^a^ | 0.08±0.02^a^ | — |
|  | F6 | Octadecanoic acid | 1957/11/4 | C_1_8H_36_O_2_ | 284.3 | 2343 | 31.67 | — | 0.03±0.01^a^ | — | — | — |
| **Alkanes** | G1 | Methylene chloride | 1975/9/2 | CH_2_Cl_2_ | 84 | 937 | 4.7 | 4.07±0.04^b^ | 2.22±0.03^c^ | 4.43±0.09^a^ | 3.98±0.04^b^ | — |
|  | G2 | Pentane | 109-66-0 | C_5_H_12_ | 72.1 | 584 | 1.17 | — | — | 0.01±0.01^a^ | — | — |
|  | G3 | n-Hexane | 110-54-3 | C_6_H_14_ | 86.1 | 640 | 1.16 | 1.13±0.03^a^ | 0.02±0.02^c^ | 0.02±0.01^c^ | 0.69±0.04^b^ | — |
|  | G4 | Methyltris(trimethylsiloxy)silane | 17928-28-8 | C_10_H_30_O_3_Si_4_ | 310.1 | 1116 | 10.1 | 0.13±0.02^a^ | — | 0.17±0.04^a^ | 0.12±0.03^a^ | — |
|  | G5 | Cyclopentasiloxane, decamethyl- | 5989-27-5 | C_10_H_16_ | 136.1 | 1200 | 12.53 | — | 0.26±0.02^a^ | — | — | — |
|  | G6 | Tridecane | 629-50-5 | C_13_H_28_ | 184.2 | 1299 | 15.75 | — | — | 0.02±0.01^a^ | 0.01±0.01^a^ | — |
|  | G7 | (*Z*)-2,2-Dimethyl-3-(3-methylpenta-2,4-dien-1-yl)oxirane | 33281-83-3 | C_10_H_16_O | 152.1 | 1472 | 20.24 | 0.12±0.03^a^ | 0.06±0.02^b^ | — | — | — |
|  | G8 | Pentadecane | 629-62-9 | C_15_H_32_ | 212.3 | 1499 | 20.85 | — | — | 0.05±0.01^a^ | — | — |
|  | G9 | Bicyclo[3.2.1]oct-2-ene3-methyl-4-methylene- | 49826-53-1 | C_10_H_14_ | 134.1 | 1356 | 17.34 | — | 0.01±0.01^a^ | — | — | — |
|  | G10 | Kessane | 3321-66-2 | C_15_H_26_O | 222.2 | 1785 | 25.46 | 0.02±0.01^b^ | 0.12±0.06^a^ | 0.03±0.03^b^ | — | — |
| **Others** | H1 | Methyl eugenol | 93-15-2 | C_11_H_14_O_2_ | 178.1 | 2028 | 28.26 | 0.35±0.04^b^ | 0.05±0.01^c^ | 0.40±0.05^b^ | 0.89±0.05^a^ | — |
|  | H2 | Eugenol | 97-53-0 | C_10_H_12_O_2_ | 164.1 | 2195 | 29.97 | — | — | 0.03±0.01^b^ | 0.06±0.02^a^ | — |
|  | H3 | 2,4-Di-tert-butylphenol | 96-76-4 | C_14_H_22_O | 206.2 | 2308 | 31.23 | — | — | 0.02±0.01^a^ | — | — |
|  | H4 | Elemicin | 487-11-6 | C_12_H_16_O_3_ | 208.1 | 2241 | 30.48 | 2.32±0.06^a^ | 0.29±0.05^c^ | 1.19±0.05^b^ | 0.22±0.02^c^ | — |
|  | H5 | Hydrazinecarboxamide | 57-56-7 | CH_5_N_3_O | 75 | 637 | 0.9 | 0.21±0.03^a^ | 0.08±0.01^b^ | 0.23±0.04^a^ | 0.26±0.02^a^ | — |
|  | H6 | Dimethyl sulfide | 75-18-3 | C_2_H_6_S | 62 | 766 | 1.89 | 0.01±0.01^b^ | 0.02±0.01^b^ | 0.03±0.02^b^ | 0.07±0.01^a^ | sulfurous |
|  | H7 | Methyl isocyanide | 593-75-9 | C_2_H_3_N | 41 | 1016 | 6.83 | 0.08±0.02^a^ | 0.06±0.03^a^ | 0.12±0.04^a^ | 0.06±0.04^a^ | — |
|  | H8 | 2,3-Dihydrofuran | 1191-99-7 | C_4_H_6_O | 70 | 1059 | 8.21 | — | — | 0.05±0.03^a^ | — | — |
|  | H9 | Ethylbenzene | 100-41-4 | C_8_H_10_ | 106.1 | 1131 | 10.51 | 0.02±0.02^a^ | — | — | — | — |
|  | H10 | p-Xylene | 106-42-3 | C_8_H_10_ | 106.1 | 1148 | 11.06 | — | 0.04±0.02^a^ | — | — | — |
|  | H11 | Albene | 38451-64-8 | C_12_H_18_ | 162.1 | 1312 | 16.11 | — | — | — | 0.02±0.01^a^ | — |
|  | H12 | Benzene, 2-ethenyl-1,3-dimethyl- | 2039-90-9 | C_10_H_12_ | 132.1 | 1453 | 19.79 | 0.13±0.02^a^ | 0.09±0.04^a^ | — | 0.12±0.06^a^ | — |
|  | H13 | Benzene, 1,2-dichloro | 541-73-1 | C_6_H_4_Cl_2_ | 146 | 1500 | 20.88 | 0.07±0.02^a^ | 0.01±0.01^b^ | — | — | — |
|  | H14 | Pyrazine, 2-methoxy-3-(1-methylpropyl)- | 24168-70-5 | C_9_H_14_N_2_O | 166.1 | 1456 | 20.96 | — | 0.02±0.01^a^ | — | — | — |
|  | H15 | Estragole | 140-67-0 | C_10_H_12_O | 148.1 | 1683 | 24.03 | 0.33±0.05^b^ | — | — | 25.12±0.05^a^ | — |
|  | H16 | Octanoic anhydride | 623-66-5 | C_16_H_30_O_3_ | 270.2 | 1847 | 26.21 | 0.23±0.04^a^ | 0.08±0.01^b^ | — | 0.20±0.02^a^ | — |
|  | H17 | Anethole | 104-46-1 | C_10_H_12_O | 148.1 | 1854 | 26.3 | 0.12±0.03^b^ | — | — | 0.45±0.05^a^ | — |
|  | H18 | Caryophyllene oxide | 1139-30-6 | C_15_H_24_O | 220.2 | 2023 | 28.21 | — | 0.05±0.03^b^ | 0.09±0.02^a^ | — | — |
|  | H19 | Cyclohexano-15-crown-5 | 17454-48-7 | C_14_H_26_O_5_ | 274.2 | 2050 | 28.49 | — | 0.01±0.01^a^ | — | — | — |
|  | H20 | Myroxide | 28977-57-3 | C_10_H_16_O | 152.1 | 1492 | 20.7 | 0.31±0.05^a^ | 0.13±0.02^b^ | 0.30±0.03^a^ | 0.30±0.06^a^ | — |
|  | H21 | 4-Ethylbenzamide | 33695-58-8 | C_9_H_11_NO | 149.1 | 1204 | 12.85 | — | — | 0.02±0.01^a^ | 0.03±0.02^a^ | — |
| Different letters within the same row for different ultraviolet irradiation conditions of significant differences at a significance level of p<0.05.“—”represents non-detection of the VOCs. Odor description from: https://www.femaflavor.org/flavor-library. | | | | | | | | | | | | |

**Table S3.** Comparative analysis of co-detected compounds by HS-GC-IMS and HS-SPME-GC-MS.

| **Category** | **NO** | **Chemicals** | **Methods analysis** | **Ion aggregation states** | **Cas** | **Formula** | **Molecular** | **Retention index（RI）** | **Retention time/s** | **Drift time** | **Relative content（%）** | | | | **Odor description** |
| --- | --- | --- | --- | --- | --- | --- | --- | --- | --- | --- | --- | --- | --- | --- | --- |
|  |  |  |  |  |  |  | **weight** |  |  | **/ms** | **DZ** | **UV-A** | **UV-B** | **UV-A+UV-B** |  |
| Terpenes | 1 | (R/S)-Limonene | HS-GC-IMS | D | 138-86-3 | C_10_H_16_ | 136.2 | 1035 | 664.08 | 1.22 | 2.82±0.10^a^ | 1.54±0.05^c^ | 2.32±0.11^b^ | 2.24±0.04^b^ | lemon, sweet, orange, pine oil |
|  |  |  | HS-GC-IMS | M | — | — | — | 1035 | 664.59 | 1.29 | 1.72±0.06^ab^ | 1.52±0.01^c^ | 1.77±005^a^ | 1.65±0.07^b^ |  |
|  |  |  | HS-GC-IMS | P | — | — | — | 1034 | 662.54 | 1.65 | 1.31±0.03^a^ | 0.80±0.02^b^ | 0.87±0.13^b^ | 1.27±0.08^a^ |  |
|  |  |  | HS-GC-IMS | P | — | — | — | 1034 | 662.54 | 1.73 | 1.32±0.04^a^ | 0.78±0.02^b^ | 0.85±0.13^b^ | 1.30±0.13^a^ |  |
|  |  |  | HS-SPME-GC-MS | — | — | — | — | 1200 | 12.72 | — | 5.24±0.06^a^ | 0.26±0.10^d^ | 1.36±0.04^c^ | 1.56±0.13^b^ |  |
|  | 2 | *α*-Pinene | HS-GC-IMS | D | 80-56-8 | C_10_H_16_ | 136.2 | 930 | 442.26 | 1.21 | 1.40±0.02^a^ | 1.09±0.02^d^ | 1.32±0.01^b^ | 1.27±0.02^c^ | Fruity, fresh, camphor, sweet, pine wood |
|  |  |  | HS-GC-IMS | M | — | — | — | 931 | 444.24 | 1.29 | 2.59±0.05^b^ | 2.29±0.02^c^ | 2.75±0.01^a^ | 2.58±0.08^b^ |  |
|  |  |  | HS-GC-IMS | P | — | — | — | 932 | 445.23 | 1.67 | 3.58±0.07^a^ | 2.50±0.06^b^ | 2.62±0.23^b^ | 3.33±0.11^a^ |  |
|  |  |  | HS-GC-IMS | P | — | — | — | 930 | 443.25 | 1.73 | 1.23±0.03^a^ | 0.89±0.02^b^ | 0.92±0.07^b^ | 1.14±0.08^a^ |  |
|  |  |  | HS-SPME-GC-MS | — | — | — | — | 1021 | 6.98 | — | 1.31±0.04^a^ | 0.09±0.03^c^ | 0.37±0.01^b^ | 0.37±0.03^b^ |  |
|  | 3 | Terpinolene | HS-GC-IMS | — | 586-62-9 | C_10_H_16_ | 136.2 | 1086 | 761.42 | 1.22 | 1.12±0.03^a^ | 0.78±0.01^c^ | 0.52±0.04^d^ | 1.03±0.07^b^ | fresh woody sweet pine citrus |
|  |  |  | HS-SPME-GC-MS | — | — | — | — | 1280 | 15.26 | — | 0.03±0.01^c^ | 0.93±0.04^b^ | 5.93±0.08^a^ | 0.08±0.02^c^ |  |
|  | 4 | (*E*)-*β*-ocimene | HS-GC-IMS | D | 13877-91-3 | C_10_H_16_ | 136.2 | 1048 | 657.08 | 1.72 | 4.21±0.08^b^ | 3.91±0.05^d^ | 4.44±0.06^a^ | 4.01±0.10^c^ | floral, woody, fruity |
|  |  |  | HS-GC-IMS | P | — | — | — | 1047 | 655.30 | 2.18 | 1.45±0.04^b^ | 1.64±0.06^a^ | 1.46±0.08^b^ | 1.50±0.04^b^ |  |
|  |  |  | HS-SPME-GC-MS | — | — | — | — | 1252 | 14.47 | — | 24.32±0.03^b^ | 38.76±0.31^a^ | 17.25±0.06^c^ | 14.29±0.10^d^ |  |
|  | 5 | *β*-Pinene | HS-GC-IMS | D | 127-91-3 | C_10_H_16_ | 136.2 | 974 | 522.77 | 1.22 | 0.92±0.01^d^ | 1.26±0.03^b^ | 1.81±0.07^a^ | 1.06±0.02^c^ | resin, green |
|  |  |  | HS-GC-IMS | M | — | — | — | 974 | 523.26 | 1.29 | 3.87±0.06^b^ | 3.39±0.05^c^ | 4.10±0.11^a^ | 3.85±0.18^b^ |  |
|  |  |  | HS-GC-IMS | P | — | — | — | 975 | 524.25 | 1.65 | 1.91±0.05^d^ | 3.00±0.04^b^ | 3.14±0.01^a^ | 2.66±0.05^c^ |  |
|  |  |  | HS-GC-IMS | P | — | — | — | 975 | 523.75 | 1.73 | 1.39±0.06^c^ | 2.47±0.05^a^ | 2.40±0.03^a^ | 1.95±0.05^b^ |  |
|  |  |  | HS-GC-IMS | P | — | — | — | 974 | 521.78 | 2.16 | 1.28±0.04^d^ | 4.89±0.09^a^ | 3.50±0.14^b^ | 2.14±0.21^c^ |  |
|  |  |  | HS-SPME-GC-MS | — | — | — | — | 1105 | 9.66 | — | 0.62±0.04^a^ | 0.05±0.02^c^ | 0.50±0.07^b^ | 0.12±0.03^c^ |  |
|  | 6 | *γ*-terpinene | HS-GC-IMS | D | 99-85-4 | C_10_H_16_ | 136.2 | 1057 | 677.04 | 1.70 | 5.76±0.05^c^ | 5.36±0.09^d^ | 6.61±0.06^a^ | 6.07±0.20^b^ | oil, wood, terpenes, lemon, lime, herbs |
|  |  |  | HS-GC-IMS | P | — | — | — | 1057 | 676.43 | 2.16 | 1.90±0.03^b^ | 1.72±0.02^c^ | 2.20±0.13^a^ | 1.98±0.09^b^ |  |
|  |  |  | HS-SPME-GC-MS | — | — | — | — | 1244 | 14.21 | — | 0.03±0.01^a^ | 0.01±0.01^b^ | 0.03±0.02^a^ | — |  |
|  | 7 | Myrcene | HS-GC-IMS | D | 123-35-3 | C_10_H_16_ | 136.2 | 992 | 559.31 | 1.22 | 1.98±0.08^a^ | 1.29±0.02^c^ | 1.85±0.04^b^ | 1.81±0.03^b^ | must, spice, balsamic |
|  |  |  | HS-GC-IMS | M | — | — | — | 992 | 559.81 | 1.28 | 1.80±0.05^b^ | 1.84±0.01^b^ | 2.12±0.06^a^ | 1.83±0.12^b^ |  |
|  |  |  | HS-GC-IMS | P | — | — | — | 992 | 559.31 | 1.64 | 0.98±0.01^b^ | 0.60±0.00^d^ | 0.90±0.01^c^ | 1.02±0.01^a^ |  |
|  |  |  | HS-GC-IMS | P | — | — | — | 992 | 558.82 | 1.71 | 3.52±0.05^a^ | 2.02±0.04^c^ | 3.14±0.11^b^ | 3.59±0.06^a^ |  |
|  |  |  | HS-GC-IMS | P | — | — | — | 991 | 556.35 | 2.17 | 1.39±0.06^a^ | 0.46±0.05^c^ | 0.88±0.04^b^ | 1.44±0.15^a^ |  |
|  |  |  | HS-SPME-GC-MS | — | — | — | — | 1166 | 11.64 | — | 3.14±0.02^a^ | 1.14±0.12^d^ | 1.73±0.17^b^ | 1.40±0.10^c^ |  |
| Aldehydes | 8 | Propanal | HS-GC-IMS | — | 123-38-6 | C_3_H_6_O | 58.1 | 500 | 113.14 | 1.05 | 1.25±0.03^a^ | 0.91±0.07^c^ | 1.25±0.03^a^ | 1.11±0.03^b^ | pungent, green grassy |
|  |  |  | HS-SPME-GC-MS | — | — | — | — | 812 | 2.35 | — | — | — | 0.02±0.02^a^ | — |  |
| Ketones | 9 | Acetone | HS-GC-IMS | — | C67641 | C_3_H_6_O | 58.1 | 533 | 117.28 | 1.11 | 7.60±0.09^a^ | 5.69±0.05^d^ | 7.21±0.13^b^ | 6.89±0.28^c^ | Fresh, apple, pear |
|  |  |  | HS-SPME-GC-MS | — | — | — | — | 831 | 2.66 | — | 0.17±0.02^b^ | 0.17±0.03^b^ | 0.28±0.04^a^ | 0.19±0.01^b^ |  |
| Others | 10 | Diethyl sulfide | HS-GC-IMS | — | C352932 | C_4_H_10_S | 90.2 | 702 | 199.52 | 1.05 | 0.17±0.01^b^ | 0.35±0.02^a^ | 0.16±0.01^b^ | 0.12±0.00^c^ | sulfurous |
|  |  |  | HS-SPME-GC-MS | — | — | — | — | 766 | 1.89 | — | 0.01±0.01^b^ | 0.02±0.01^b^ | 0.03±0.02^b^ | 0.07±0.01^a^ |  |
| The ion aggregation states M, D, and P refer to monomer, dimer, and polymer, respectively. Different letters within the same row for different ultraviolet irradiation conditions of significant differences at a significance level of *p*<0.05.“—”represents non-detection of the VOCs. Odor description from: https://www.femaflavor.org/flavor-library. | | | | | | | | | | | | | | | |

**Table S4.** Selected compounds with VIP score >1 from HS-GC-IMS and HS-SPME-GC-MS with OPLS-DA models for significant classification of different ultraviolet irradiation conditions.

| **Category** | **Compounds** | **HS-GC-IMS** | **VIP** | **HS-SPME-GC-MS** | **VIP** |
| --- | --- | --- | --- | --- | --- |
|  |  |  |  |  |  |
| Terpenes | 1 | *β*-Pinene | 1.00 | *β*-Pinene | 1.12 |
|  | 2 | (*E*)-*β*-ocimene | 1.04 | (*E*)-*β*-ocimene | 1.07 |
|  | 3 | — | — | *α*-Pinene | 1.12 |
|  | 4 | — | — | Camphene | 1.10 |
|  | 5 | — | — | *α*-Phellandrene | 1.06 |
|  | 6 | — | — | Myrcene | 1.13 |
|  | 7 | — | — | Limonene | 1.12 |
|  | 8 | — | — | *β*-Phellandrene | 1.06 |
|  | 9 | — | — | *trans*-*β*-Ocimene | 1.10 |
|  | 10 | — | — | *γ*-terpinene | 1.01 |
|  | 11 | — | — | 3-Carene | 1.03 |
|  | 12 | — | — | Terpinolene | 1.04 |
|  | 13 | — | — | 1,3,5,7-Cyclooctatetraene | 1.11 |
|  | 14 | — | — | (*E*)-4,8-Dimethylnona-1,3,7-triene | 1.03 |
|  | 15 | — | — | Pseudolimonene | 1.02 |
|  | 16 | — | — | Pentamethylcyclopentadiene | 1.06 |
|  | 17 | — | — | 2,4,6-Octatriene, 2,6-dimethyl-, (*E,Z*)- | 1.07 |
|  | 18 | — | — | Benzene, 1-ethenyl-3,5-dimethyl- | 1.03 |
|  | 19 | — | — | *δ*-elemene | 1.05 |
|  | 20 | — | — | *α*-Farnesene | 1.03 |
|  | 21 | — | — | (-)-*β*-Bourbonene | 1.03 |
|  | 22 | — | — | (-)-alpha-cedrene | 1.03 |
|  | 23 | — | — | *trans*-*α*-Bergamotene | 1.13 |
|  | 24 | — | — | Aristolene | 1.10 |
|  | 25 | — | — | *β*-Patchoulene | 1.03 |
|  | 26 | — | — | *β*-Caryophyllene | 1.08 |
|  | 27 | — | — | B-Cedrene | 1.04 |
|  | 28 | — | — | Bicyclosesquiphellandrene | 1.03 |
|  | 29 | — | — | (+)-Prezizaene | 1.03 |
|  | 30 | — | — | (*E*)-*β*-Farnesene | 1.03 |
|  | 31 | — | — | *γ*-Muurolene | 1.05 |
|  | 32 | — | — | *α*-Humulene | 1.06 |
|  | 33 | — | — | *α*-*cis*-Bergamotene | 1.11 |
|  | 34 | — | — | Germacrene D | 1.09 |
|  | 35 | — | — | *α*-amorphene | 1.03 |
|  | 36 | — | — | bicyclogermacrene | 1.03 |
|  | 37 | — | — | *β*-Cadinene | 1.03 |
|  | 38 | — | — | *β*-Copaene | 1.06 |
|  | 39 | — | — | Hotrienol | 1.02 |
|  | 40 | — | — | *α*-Bisabolene | 1.06 |
|  | 41 | — | — | *β*-sesquiphellandrene | 1.13 |
|  | 42 | — | — | Dehydrocycloisolongifolen | 1.10 |
|  | 43 | — | — | (+)-Limonene oxide | 1.06 |
|  | 44 | — | — | (+)-4*α*-hydroxy-2-carene | 1.06 |
|  | 45 | — | — | *β*-Maaliene | 1.01 |
|  | 46 | — | — | o-Cymene | 1.03 |
|  | 47 | — | — | 1,4-Dimethylazulene | 1.08 |
| Alcohols | 1 | 3-Methylbutanol | 1.21 | 2-Phenyl-1,3-propanediol | 1.13 |
|  | 2 | (*Z*)-2-Pentenol | 1.03 | 2-Methylbenzyl Alcohol | 1.02 |
|  | 3 | Dihydrolinalool | 1.06 | 10-Undecyn-1-ol | 1.06 |
|  | 4 | 1-Menthol | 1.12 | *trans*,*cis*-2,6-Nonadien-1-ol | 1.00 |
|  | 5 | 1-Hexanol | 1.02 | 1-Octen-3-ol | 1.02 |
|  | 6 | 1-Menthol | 1.00 | Linalool | 1.03 |
|  | 7 | — | — | (-)-*trans*-pinocarveol） | 1.00 |
|  | 8 | — | — | Coniferyl alcohol | 1.02 |
|  | 9 | — | — | Spathulenol | 1.06 |
|  | 10 | — | — | Isospathulenol | 1.02 |
| Aldehydes | 1 | (*E*)-2-Heptenal | 1.00 | Hexanal | 1.10 |
|  | 2 | 2-Methyl butanal | 1.12 | 2,6-Nonadienal, 3,7-dimethyl | 1.12 |
|  | 3 | Heptanal | 1.05 | Piperidine-1-carbaldehyde | 1.01 |
|  | 4 | — | — | Citronellal | 1.02 |
|  | 5 | — | — | 2,3-expoxycitral | 1.05 |
| Ketones | 1 | 2.3-Butanedione | 1.04 | 1,5-Heptadien-4-one, 3,3,6-trimethyl- | 1.06 |
|  | 2 | 6-Methyl. 5 hepten-2-one | 1.03 | 2-Methyl-6-methyleneocta-2,7-dien-4-one | 1.05 |
|  | 3 | 2-Butanone | 1.02 | *trans*,*trans*-3,5-Heptadien-2-one | 1.01 |
|  | 4 | Acetone | 1.00 | *γ*-himachalene | 1.04 |
|  | 5 | — | — | 4-(2,2-Dimethyl-6-methylenecyclohexylidene)-3- methylbutan-2-one | 1.12 |
|  | 6 | — | — | Benzophenone | 1.01 |
|  | 7 | — | — | Capillin | 1.12 |
|  | 8 | — | — | Geranylacetone | 1.01 |
| Acids | 1 | Benzoic acid | 1.04 | 1-Hexanoic acid | 1.03 |
|  |  | — | — | Pterin-6-carboxylic acid | 1.04 |
| Esters | 1 | Isoamyl acetate | 1.26 | Octaethylene glycol monododecyl ether | 1.04 |
|  | 2 | Acetic acid pentyl ester | 1.22 | Propanoic acid, butyl ester | 1.02 |
|  | 3 | Butyl acetate | 1.13 | *cis*-3-Hexenyl isovalerate | 1.03 |
|  | 4 | Prenyl acetate | 1.00 | Methyl dihydrojasmonate | 1.05 |
|  | 5 | — | — | Ethyl (*E*)-cinnamate | 1.04 |
|  | 6 | — | — | gamma-Decanolactone | 1.01 |
|  | 7 | — | — | Borneol acetate | 1.12 |
|  | 8 | — | — | *γ*-Decalactone | 1.04 |
| Alkanes | 1 | — | — | Methylene chloride | 1.07 |
|  | 2 | — | — | n-Hexane | 1.09 |
|  | 3 | — | — | Methyltris(trimethylsiloxy)silane | 1.03 |
|  | 4 | — | — | Cyclopentasiloxane, decamethyl- | 1.08 |
|  | 5 | — | — | (*Z*)-2,2-Dimethyl-3-(3-methylpenta-2,4-dien-1-yl)oxirane | 1.08 |
|  | 6 | — | — | Pentadecane | 1.03 |
|  | 7 | — | — | Bicyclo[3.2.1]oct-2-ene, 3-methyl-4-methylene- | 1.02 |
|  | 8 | — | — | Kessane | 1.00 |
| Others | 1 | 1.4-Cineol | 1.08 | Methyl eugenol | 1.10 |
|  | 2 | — | — | Eugenol | 1.07 |
|  | 3 | — | — | Elemicin | 1.14 |
|  | 4 | — | — | Hydrazinecarboxamide | 1.04 |
|  | 5 | — | — | Dimethyl sulfide | 1.08 |
|  | 6 | — | — | Albene | 1.05 |
|  | 7 | — | — | Benzene, 1,2-dichloro | 1.10 |
|  | 8 | — | — | Pyrazine, 2-methoxy-3-(1-methylpropyl)- | 1.04 |
|  | 9 | — | — | Estragole | 1.13 |
|  | 10 | — | — | Octanoic anhydride | 1.06 |
|  | 11 | — | — | Anethole | 1.11 |
|  | 12 | — | — | Caryophyllene oxide | 1.03 |
|  | 13 | — | — | Cyclohexano-15-crown-5 | 1.02 |
